# Best-first search–based approach for mining top-k closed frequent itemsets from uncertain databases

**DOI:** 10.1371/journal.pone.0351951

**Published:** 2026-06-17

**Authors:** Nguyen Le, Huy Vo, Thien Nguyen

**Affiliations:** 1 Faculty of Information Technology, Ton Duc Thang University, Ho Chi Minh City, Vietnam; 2 Natural Language Processing and Knowledge Discovery Research Group, Faculty of Information Technology, Ton Duc Thang University, Ho Chi Minh City, Vietnam; The Kyoto College of Graduate Studies for Informatics: Kyoto Joho Daigakuin Daigaku, JAPAN

## Abstract

Uncertain data mining has become critical due to data generated by sensor networks, RFID systems, and data integration platforms. Mining top-k closed frequent itemsets from uncertain databases is particularly challenging because probabilistic support evaluation is expensive and the search space grows exponentially. Most existing methods rely on depth-first search (DFS) traversal, which explores candidates in enumeration order and often discovers high-support patterns late, leading to weak pruning and costly closure verification. This paper proposes TUFCI, a best-first-search-based algorithm for mining top-k closed frequent itemsets from uncertain databases. TUFCI explores candidates in descending order of probabilistic support using a priority queue, enabling early discovery of strong patterns, rapid threshold elevation, and safe early termination. Support-ordered exploration also improves closure checking by prioritizing supersets most likely to violate the closure property, thereby reducing redundant superset examinations. Experimental results demonstrate that TUFCI significantly outperforms DFS-based approaches in runtime and reduces the number of closure checks, especially on dense datasets.

## Introduction

Frequent itemset mining has been a fundamental task in data mining since the Apriori algorithm was introduced by Agrawal and Srikant [1]. Luna et al. [2] survey more than two decades of progress in this field. Traditional algorithms assume deterministic item occurrences, which is unrealistic for many modern data sources such as sensor networks, data integration systems, and privacy-preserving applications [3]. To address this issue, Aggarwal and Yu [4] established the foundations of uncertain data management.

The exponential growth of data and the combinatorial explosion of candidate patterns make pattern mining increasingly expensive. Closed frequent itemsets provide a compact and lossless representation of frequent patterns [5], while the top-k formulation avoids the difficult task of selecting an appropriate minimum support threshold by allowing users to specify only the desired number of patterns [6]. These properties are particularly important for uncertain databases, where computing probabilistic support is costly and pruning is essential.

Most existing algorithms for top-k closed itemset mining adopt depth-first search (DFS) traversal because of its low memory usage [7,8]. However, DFS explores candidates in enumeration order determined by item indexing, without considering their probabilistic support values [8,9]. As a result, high-support patterns are often discovered late, delaying the increase of the dynamic pruning threshold and weakening pruning effectiveness. Moreover, closure verification—which requires checking whether any direct superset has the same support—becomes expensive under DFS because supersets are examined in arbitrary order rather than by their likelihood of violating the closure property. This leads to redundant superset examinations and unnecessary computation when high-support patterns appear in late-explored branches.

Prior work on uncertain frequent itemset mining has mainly focused on probabilistic support computation and pruning strategies [4,10], including optimizations based on generating functions, upper bounds, and efficient data structures [3,11,12]. In contrast, the impact of search order itself on pruning efficiency and closure verification has received limited attention. Priority-queue-guided search has been explored in high-utility itemset mining (e.g., TKO [13], kHMC [14]) and in deterministic top-*k* pattern mining [6]. However, these methods operate on utility functions or deterministic support—neither of which involves the $O(m_{X}^{2})$ probabilistic support computation that dominates cost in uncertain databases. To the best of our knowledge, TUFCI is the first algorithm to combine best-first search with closure checking for top-*k* frequent closed itemset mining over uncertain data. The key technical distinction is that the generating-function convolution cost ($O(m_{X}^{2})$ per candidate, Section “Probabilistic Support Evaluation”) makes traversal order far more consequential than in deterministic or utility-based settings where support or utility is computed in $O(m_{X})$.

This paper proposes a best-first search strategy for mining top-k closed frequent itemsets from uncertain databases. Candidates are explored in descending order of probabilistic support using a priority queue, enabling early discovery of strong patterns, rapid threshold elevation, and safe early termination. Support-ordered exploration also improves closure checking by examining supersets most likely to violate the closure property first, allowing violations to be detected early and reducing redundant superset examinations.

The main contributions of this paper are as follows:

- We propose a best-first search framework for mining top-k closed frequent itemsets from uncertain databases, where candidates are processed in descending order of probabilistic support.
- We introduce a closure verification strategy that exploits support-ordered exploration to reduce redundant superset checks and improve efficiency.
- We develop TUFCI, an algorithm integrating multiple pruning techniques designed specifically for best-first traversal.
- We conduct extensive experiments demonstrating that the proposed approach achieves substantial runtime improvements over DFS-based methods, with results validated across dense and sparse datasets using external baselines and internal ablation studies.

## Literature review

### Frequent itemset mining in deterministic databases

Frequent itemset mining (FIM) was first introduced by Agrawal et al. [1] through the Apriori algorithm. Han et al. [9] proposed FP-Growth, which avoids candidate generation via a pattern-growth paradigm, and Eclat [11] introduced a vertical-format intersection approach. These methods established the algorithmic foundations of deterministic pattern mining.

Closed frequent itemsets provide a lossless compression of the frequent pattern set [5]. Wang et al. [7] proposed CLOSET+ with pruning optimizations, and the TFP algorithm [6] extended closure-based mining to the top-*k* setting by dynamically raising the support threshold. These methods rely on depth-first traversal, where exploration order is determined by item indexing rather than support values.

### Frequent itemset mining in uncertain databases

Uncertain databases assign existential probabilities to items, making support evaluation probabilistic rather than deterministic. Chui et al. [8] pioneered this area with U-Apriori, and Bernecker et al. [3] formalised probabilistic FIM under possible-world semantics. Generating functions have since become the standard approach for computing probabilistic support distributions [12]. Sun et al. [12] extended closed itemset mining to uncertain databases, and Ahmed et al. [15] explored evolutionary approaches. However, these studies focus on probabilistic modeling and computation efficiency, while the impact of search traversal order on closure verification and dynamic threshold convergence remains largely unexplored.

### High-utility and top-*k* itemset mining

High-utility itemset mining (HUIM) extends FIM by incorporating item quantities and profits [16]. Singh and Biswas [17] survey top-*k* HUIM algorithms. Tseng et al. [13] proposed TKO as a single-phase approach, while Duong et al. [14] introduced threshold-raising strategies (RIU, CUD, COV). Although utility mining differs from uncertain FIM in its objective function and monotonicity properties, both domains share the challenge of delayed threshold convergence under depth-first traversal, including recent works in the field [18,19].

### Search strategies in pattern mining

Priority-queue-guided exploration has been adopted in high-utility mining (TKO [13], kHMC [14]) and deterministic top-*k* pattern mining [6]. However, uncertain FIM has predominantly relied on level-wise or pattern-growth enumeration [8,12]. The computational bottleneck unique to uncertain databases—$O(m_{X}^{2})$ polynomial convolution per candidate—is absent in deterministic and utility-based settings, where support or utility is computable in $O(m_{X})$. This cost asymmetry makes traversal order more consequential in the uncertain setting and motivates the best-first framework proposed in this paper.

To sharpen the novelty claim, Table 1 summarises how TUFCI differs from the most closely related prior frameworks along four technical dimensions.

**Table 1:** Comparison of TUFCI with related best-first and branch-and-bound frameworks.

Method	Data model	Per-candidate cost	Closure	Early termination
TKO [13]	Deterministic	$O(m_{X})$ utility	No	Threshold-based
kHMC [14]	Deterministic	$O(m_{X})$ utility	No	Threshold-based
TFP [6]	Deterministic	$O(m_{X})$ support	Yes	Threshold-based
TopKPFIM [20]	Uncertain	$O(m_{X}^{2})$ conv.	No	None
ITUFP [21]	Uncertain	$O(m_{X}^{2})$ conv.	No	Interactive only
**TUFCI (ours)**	Uncertain	$O(m_{X}^{2})$ conv.	**Yes**	**Support-ordered**

Three distinctions separate TUFCI from prior best-first work. First, TKO and kHMC operate on *deterministic* utility functions computable in $O(m_{X})$; the $O(m_{X}^{2})$ Poisson-binomial convolution in TUFCI makes traversal order far more consequential per candidate, as a single skipped convolution saves quadratically more work. Second, neither TKO nor kHMC incorporates *closure checking*: they mine top-*k* itemsets, not top-*k* closed itemsets, so their priority queues need not interact with a closure verification module. TUFCI’s support-ordered closure verification (Theorem 2) is a novel component that exploits the same priority ordering to reduce superset examinations. Third, TopKPFIM and ITUFP operate on uncertain data but use depth-first traversal and have no early termination guarantee; TUFCI’s Lemma 5 provides a provably safe stopping condition absent in those methods.

In summary, existing work has extensively investigated probabilistic support computation, pruning strategies, and compact pattern representations. Priority-based exploration has been adopted in utility mining but not in uncertain frequent itemset mining, where the dominant cost structure (polynomial convolution) differs fundamentally. The influence of search order on closure verification efficiency and dynamic threshold convergence has received limited attention in the uncertain setting. This gap motivates TUFCI: a best-first search framework that aligns the exploration order with the top-*k* objective, specifically targeting the high per-candidate cost of probabilistic support evaluation.

### Problem statement and related definitions

For clarity, Table 4 summarises the notation used throughout this paper. The algorithm is referred to exclusively as TUFCI (Top-*k* Uncertain Frequent Closed Itemset mining). We reserve lowercase Greek letters for thresholds and probabilities (τ, θ, σ), calligraphic letters for collections and data structures (𝒟, ℋ, 𝒬), and adopt two shorthand conventions: sup(·) for ${ProbSup}_{\tau }(\cdot )$ when threshold τ is clear, and σ for θ(ℋ) when the heap context is clear from context.

### Uncertain database model

We adopt the tuple-level independence assumption commonly used in probabilistic data mining. Under this assumption, the occurrence of each item in a transaction is modeled as an independent Bernoulli random variable, and item existence events are independent across both items and transactions.

**Definition 1** (Itemset). *Let* $J=\{x_{1},x_{2},\ldots ,x_{m}\}$ *denote the universe of items. An itemset X is a non-empty subset of J, i.e.,* X⊆J *and* X≠∅*. Its cardinality is denoted by |X|.*

**Definition 2** (Uncertain transaction). *An uncertain transaction T over J is defined as a set of item–probability pairs*


$$\begin{array}{c} T=\{(x,p)|x\in J,\;p\in (0,1]\}, \end{array}$$ (1)

*where P(x,T)=p denotes the probability that item x appears in T. Items with P(x,T)=0 are considered absent.*

**Definition 3** (Uncertain database). *An uncertain database is a collection of N uncertain transactions,*


$$\begin{array}{c} 𝒟=\{T_{1},T_{2},\ldots ,T_{N}\}. \end{array}$$ (2)

*For convenience,* $P(x,T_{i})$ *denotes the existence probability of item x in transaction* $T_{i}$*, and* $P(x,T_{i})=0$ *if* $x\notin T_{i}$.

Under the tuple-level independence assumption, for any transaction $T_{i}$ and any itemset X⊆J, the probability that *X* appears in $T_{i}$ is


$$\begin{array}{c} P(X\subseteq T_{i})=\prod_{x\in X}P(x,T_{i}). \end{array}$$ (3)

**Example 1.** *Consider the uncertain database* 𝒟 *in* Table 2*, where J={A,B,C,D,E}.*

**Table 2:** An uncertain database.

TID	Uncertain transaction ($T_{i}$)
*T*_1_	{(*A*,0.9),(*B*,0.8),(*C*,0.7)}
*T*_2_	{(*A*,0.8),(*B*,0.7),(*D*,0.6)}
*T*_3_	{(*A*,0.9),(*C*,0.8),(*D*,0.7)}
*T*_4_	{(*B*,0.8),(*C*,0.9),(*E*,0.5)}
*T*_5_	{(*A*,0.7),(*B*,0.6),(*C*,0.8),(*D*,0.9)}

For itemset *X*={*A*,*B*}, the probability that *X* appears in transaction *T*_1_ is


$$\begin{array}{c} P(\{A,B\}\subseteq T_{1})=P(A,T_{1})\times P(B,T_{1})=0.9\times 0.8=0.72. \end{array}$$ (4)

Since *P*(*A*,*T*_4_)=0, we obtain


$$\begin{array}{c} P(\{A,B\}\subseteq T_{4})=0. \end{array}$$ (5)

### Probabilistic support

The goal of this subsection is to define the *probabilistic support* ${ProbSup}_{\tau }(X)$, the central quantity used throughout the paper for ranking and pruning. Because item occurrences are uncertain, support is no longer a simple count but a random variable. We build up to the final definition in three steps, each motivated by a concrete need: (1) we define support as a random variable (Definition 4) to capture the inherent uncertainty; (2) we introduce generating functions as the only computationally tractable way to obtain the full distribution of that random variable (Eq. 9–10), since a direct enumeration of all $2^{N}$ possible outcomes is infeasible; and (3) we define the tail probability $F_{X}(s)$ (Definition 5) and use it to extract a single, threshold-qualified support value ${ProbSup}_{\tau }(X)$ (Definition 6) that behaves analogously to the deterministic support count and admits the same pruning arguments. Example 2 concretizes all three steps on the running database.

**Definition 4** (Support). *For an itemset X in an uncertain database* 𝒟*, the support of X is the random variable*


$$\begin{array}{c} Sup(X)\;=\;\sum_{i=1}^{N}1_{X\subseteq T_{i}}, \end{array}$$ (6)

*where* $1_{X\subseteq T_{i}}$ *equals 1 if* $X\subseteq T_{i}$ *and 0 otherwise.*

Under the tuple-level independence assumption each indicator $1_{X\subseteq T_{i}}$ is Bernoulli with success probability


$$\begin{array}{c} p_{i}\;=\;P(X\subseteq T_{i}). \end{array}$$ (7)

Hence Sup(X) follows a Poisson-binomial distribution.

Computing $p_{j}=P(X\subseteq T_{j})$ requires *O*(|*X*|) multiplications since


$$\begin{array}{c} p_{j}\;=\;\prod_{x\in X}P(x,T_{j}). \end{array}$$ (8)

Each transaction independently “contains” itemset *X* with probability $p_{j}$ and “excludes” it with probability $1-p_{j}$. The total support of *X* is therefore the sum of these independent Bernoulli outcomes.

Unlike the standard Binomial distribution, the Poisson-binomial distribution has a distinct success probability $p_{i}$ for each transaction, so its PMF has no closed-form expression. Computing it directly by enumerating all $2^{N}$ possible transaction subsets is exponentially expensive and infeasible for any realistic database. Generating functions provide an efficient alternative: by encoding each transaction’s contribution as a degree-1 polynomial and multiplying all *N* polynomials together, the coefficient of $z^{s}$ in the resulting product gives exactly P(Sup(X)=s).

To obtain the full distribution of Sup(X) efficiently we use generating functions. For each transaction $T_{j}$ (with $p_{j}>0$) define the polynomial


$$\begin{array}{c} g_{j}(z)\;=\;(1-p_{j})+p_{j}z, \end{array}$$ (9)

and the probability generating function


$$\begin{array}{c} G(z)\;=\;\prod_{j=1}^{N}g_{j}(z). \end{array}$$ (10)

The coefficient of $z^{s}$ in *G*(*z*) equals P(Sup(X)=s) (see [3]).

Having computed the distribution P(Sup(X)=s) for each *s*, a natural question is: *how likely is X to appear in at least s transactions?* The cumulative (tail) probability answers this question. Since each term in the sum is non-negative, this function is monotonically non-increasing in *s*—higher support thresholds are harder to meet.

**Definition 5** (Frequency function). *For* s∈{0,1,…,N} *the frequency function of X is*


$$\begin{array}{c} F_{X}(s)\;=\;P(Sup(X)\geq s)\;=\;\sum_{j=s}^{N}P(Sup(X)=j). \end{array}$$ (11)

The *probabilistic support* answers: what is the largest support level *s* that itemset *X* achieves with confidence at least τ? Because the frequency function is non-increasing, this maximum is well-defined and can be found efficiently via binary search on the frequency array.

**Definition 6** (Probabilistic support). *Let* τ∈(0,1] *be a probability threshold. The probabilistic support of X is the largest integer s such that* $F_{X}(s)\geq \tau$*:*


$$\begin{array}{c} {ProbSup}_{\tau }(X)\;=\;\max\{s\in {\mathbb{Z}}_{\geq 0}|F_{X}(s)\geq \tau \}. \end{array}$$ (12)

*If no such s exists then* ${ProbSup}_{\tau }(X)=0$.

**Example 2.** *Continuing from Example 1, let X={A}. The existence-probability vector is*


$$\begin{array}{c} {𝐩}_{A}=(0.9,\;0.8,\;0.9,\;0,\;0.7), \end{array}$$

*so* Sup({A}) *is Poisson-binomial.* Table 3 *gives the PMF and tail probabilities.*

**Table 3:** Support distribution for itemset {*A*}.

*s*	P(Sup({A})=s)	$F_{\left\{A\right\}}(s)=P(Sup(\{A\})\geq s)$
0	0.0006	1.0000
1	0.0162	0.9994
2	0.1198	0.9832
3	0.3790	0.8634
4	0.4844	0.4844
5	0.0000	0.0000

*With* τ=0.7 *we find* $F_{\left\{A\right\}}(3)=0.8634\geq 0.7$ *and F{A}(4)=0.4844 < 0.7, hence*


$$\begin{array}{c} {ProbSup}_{0.7}(\{A\})=3. \end{array}$$

### Frequent and closed itemsets

**Definition 7** (Frequent itemset). *Given a probability threshold* τ∈(0,1] *and a minimum support count* $s_{\min}\in {\mathbb{Z}}_{\geq 1}$*, an itemset X is said to be* $\left(\tau ,s_{\min}\right)$*-frequent if*


$$\begin{array}{c} {ProbSup}_{\tau }(X)\geq s_{\min}. \end{array}$$ (13)

*The set of all* $\left(\tau ,s_{\min}\right)$*-frequent itemsets in an uncertain database* 𝒟 *is denoted by* ℱ*. In top-k mining,* $s_{\min}$ *is not fixed by the user; instead, it is supplied by the dynamic threshold* σ=θ(ℋ) *derived from the result heap (Definition 11).*

Intuitively, a closed itemset is one that cannot be “extended” without losing support. If adding any single item to *X* always reduces its support, then *X* captures maximal information—no larger itemset carries the same statistical signal.

**Definition 8** (Closed frequent itemset). *A frequent itemset* X∈ℱ *is said to be closed if there exists no proper superset* Y⊃X *such that*


$$\begin{array}{c} {ProbSup}_{\tau }(Y)={ProbSup}_{\tau }(X). \end{array}$$ (14)

*The set of all closed frequent itemsets is denoted by* 𝒞⊆ℱ.

**Theorem 1** (Closure checking via immediate extensions). *An itemset X is closed if and only if, for all items* e∈J⧵X*, the probabilistic support strictly decreases when extending X by e, i.e.,*


$$\begin{array}{c} {ProbSup}_{\tau }(X\cup \{e\})<{ProbSup}_{\tau }(X). \end{array}$$ (15)

*Proof.* By the antimonotonicity of probabilistic support, adding an item to an itemset cannot increase its support. Therefore, if the probabilistic support strictly decreases for all immediate supersets of the form X∪{e} with e∈J⧵X, then no proper superset of *X* can have the same probabilistic support as *X*, and *X* is closed.

Conversely, if there exists an item e∈J⧵X such that


$$\begin{array}{c} {ProbSup}_{\tau }(X\cup \{e\})={ProbSup}_{\tau }(X), \end{array}$$

then *X* has a proper superset with equal probabilistic support and is therefore not closed. This completes the proof. □

**Example 3.** *Consider the uncertain database in* Table 2 *with probability threshold* τ=0.7*. Let X={A} be an itemset with*


$$\begin{array}{c} {ProbSup}_{0.7}(\{A\})=3. \end{array}$$

*To determine whether X is closed, we examine all immediate extensions obtained by adding one item from* J⧵X={B,C,D,E}*. From the database we obtain*


$$\begin{array}{c} {ProbSup}_{0.7}(\{A,B\})=2<3, \end{array}$$

$$\begin{array}{c} {ProbSup}_{0.7}(\{A,C\})=2<3, \end{array}$$

$$\begin{array}{c} {ProbSup}_{0.7}(\{A,D\})=1<3, \end{array}$$

$$\begin{array}{c} {ProbSup}_{0.7}(\{A,E\})=0<3. \end{array}$$

*Since the probabilistic support strictly decreases for all immediate extensions of X, it follows from Theorem 1 that the itemset {A} is closed.*

### Search space and enumeration

**Definition 9** (Set-enumeration tree). *The search space of itemsets is organized as a set-enumeration tree. Given a total order* ≺ *on the item universe J, the children of a node representing an itemset X are all itemsets of the form*


$$\begin{array}{c} X\cup \{i\}\;\text{such that}\;i≻\max_{≺}(X). \end{array}$$

*The root of the tree corresponds to the empty set.*

Depth-first search (DFS) explores the set-enumeration tree by fully expanding each branch before backtracking. Its traversal order is determined solely by the predefined order ≺ and is independent of itemset support values. Consequently, itemsets with high probabilistic support may be discovered late in the search process, which delays updates of the dynamic threshold and reduces pruning effectiveness.

In contrast, best-first search maintains a priority queue of candidate itemsets ordered by probabilistic support and always expands the currently most promising candidate. This strategy favors the early discovery of high-support itemsets and enables closure checking to be performed first on the most relevant supersets.

### Top-*k* mining formulation

**Definition 10** (Top-*k* closed frequent itemsets). *Let* 𝒞 *denote the set of all closed frequent itemsets in an uncertain database* 𝒟*. The set of top-k closed frequent itemsets is a subset* ${𝒞}_{k}\subseteq 𝒞$ *such that* $|{𝒞}_{k}|=k$ *and*


$$\begin{array}{c} \forall X\in {𝒞}_{k},\;\forall Y\in 𝒞⧵{𝒞}_{k}:\;{ProbSup}_{\tau }(X)\geq {ProbSup}_{\tau }(Y). \end{array}$$ (16)

*That is,* ${𝒞}_{k}$ *contains the k closed frequent itemsets with the largest probabilistic supports.*

#### Tie handling.

When more than *k* closed frequent itemsets share the probabilistic support of the *k*-th ranked itemset, Eq. (16) alone does not single out a unique set. TUFCI returns *exactly k* itemsets and resolves such boundary ties deterministically, using the same Candidate Priority Order applied during mining (Definition 13): among itemsets of equal probabilistic support, those with larger tail probability $F_{X}(sup(X))$ are ranked higher, and any remaining ties are broken in favour of smaller cardinality. The returned set is therefore well-defined. When fewer than *k* closed frequent itemsets exist for the given $\left(\tau ,\sigma_{\min}\right)$ constraints, TUFCI returns all of them.

**Definition 11** (Dynamic threshold). *During the mining process, a dynamic threshold* σ *is maintained. Initially,* σ=0*. Once k closed frequent itemsets have been identified,* σ *is updated to the probabilistic support of the current k-th ranked itemset. Any candidate itemset X satisfying*


$$\begin{array}{c} {ProbSup}_{\tau }(X)<\sigma \end{array}$$ (17)

*can be safely pruned from further consideration.*

### Problem statement

**Definition 12** (Problem: Top-*k* closed frequent itemset mining from uncertain databases). *Given an uncertain transaction database* 𝒟*, a probability threshold* τ∈(0,1]*, and an integer* k≥1*, return the set* ${𝒞}_{k}$ *of top-k closed frequent itemsets, where* ${𝒞}_{k}$ *is defined as in Definition 10 (i.e., the k closed frequent itemsets with largest probabilistic supports* ${ProbSup}_{\tau }(\cdot )$*).*

#### Input.

An uncertain database 𝒟 (Def. 3), a probability threshold τ, and an integer *k*.

#### Output.

The set ${𝒞}_{k}$ of size *k* containing the closed frequent itemsets with the largest ${ProbSup}_{\tau }(\cdot )$ values.

### Practical challenges

1. **Expensive support computation.** Evaluating $P(X\subseteq T_{i})$ requires *O*(|*X*|) multiplications per transaction and computing the full distribution of Sup(X) (or its tail probabilities) involves convolution operations (generating functions / Poisson-binomial computations).
2. **Late threshold elevation under DFS.** Depth-first traversal may discover high-support closed itemsets late, delaying updates of the dynamic threshold σ and reducing pruning effectiveness.
3. **Costly closure verification.** Closure checks require examining immediate supersets; under arbitrary exploration order (e.g., DFS) these checks may be repeated unnecessarily.
4. **Combinatorial explosion.** The search space grows exponentially with the number of items |*J*|, creating scalability and memory-pressure issues for candidate management.

### Key properties

**Lemma 1** (Antimonotonicity). *For any two itemsets* X⊆Y*, the probabilistic support satisfies*


$$\begin{array}{c} {ProbSup}_{\tau }(Y)\leq {ProbSup}_{\tau }(X). \end{array}$$ (18)

*Proof.* For each transaction $T_{t}$, define $p_{t}^{X}=P(X\subseteq T_{t})=\prod_{x\in X}P(x,T_{t})$ and $p_{t}^{Y}=P(Y\subseteq T_{t})=\prod_{x\in Y}P(x,T_{t})$. Since X⊆Y and all existence probabilities lie in [0,1], we have $p_{t}^{Y}\leq p_{t}^{X}$ for every *t*.

We establish the result via an explicit coupling. Let $\{U_{t}{\}}_{t=1}^{N}$ be independent Uniform(0, 1) random variables, and define


$$\begin{array}{c} B_{t}^{X}=1[U_{t}\leq p_{t}^{X}],\;B_{t}^{Y}=1[U_{t}\leq p_{t}^{Y}]. \end{array}$$

By construction, each $B_{t}^{X}\sim Bernoulli(p_{t}^{X})$ and $B_{t}^{Y}\sim Bernoulli(p_{t}^{Y})$, and variables are independent across transactions. Since $p_{t}^{Y}\leq p_{t}^{X}$, we have $B_{t}^{Y}\leq B_{t}^{X}$ almost surely for every *t*.

Let $S_{X}=\sum_{t=1}^{N}B_{t}^{X}$ and $S_{Y}=\sum_{t=1}^{N}B_{t}^{Y}$. The pointwise inequality $B_{t}^{Y}\leq B_{t}^{X}$ yields $S_{Y}\leq S_{X}$ almost surely. Therefore, for every s≥0:


$$\begin{array}{c} \mathrm{Pr}(S_{Y}\geq s)\;\leq \;\mathrm{Pr}(S_{X}\geq s). \end{array}$$

By Definition 6, ${ProbSup}_{\tau }(Z)=\max\{s:\mathrm{Pr}(S_{Z}\geq s)\geq \tau \}$. Since the tail probabilities of $S_{Y}$ are pointwise dominated by those of $S_{X}$, the maximum *s* satisfying the τ-threshold for *Y* cannot exceed that for *X*. Hence ${ProbSup}_{\tau }(Y)\leq {ProbSup}_{\tau }(X)$. □

**Lemma 2** (Safe pruning). *Let* σ *be the current dynamic threshold. If an itemset X satisfies*


$$\begin{array}{c} {ProbSup}_{\tau }(X)<\sigma , \end{array}$$ (19)

*then X and all its supersets can be safely pruned from further consideration.*

*Proof.* By Lemma 1, for any superset Y⊇X,


$$\begin{array}{c} {ProbSup}_{\tau }(Y)\leq {ProbSup}_{\tau }(X)<\sigma . \end{array}$$

Hence, neither *X* nor any of its supersets can reach the dynamic threshold and thus cannot belong to the top-*k* closed frequent itemsets. □

**Lemma 3** (Early termination under best-first search). *Assume that k closed frequent itemsets have already been found and that* σ *is the probabilistic support of the current k-th ranked itemset. If the best-first candidate X extracted from the priority queue satisfies*


$$\begin{array}{c} {ProbSup}_{\tau }(X)<\sigma , \end{array}$$ (20)

*then the search process can be terminated.*

*Proof.* All remaining candidates in the priority queue have probabilistic support no greater than that of *X*, i.e.,


$$\begin{array}{c} {ProbSup}_{\tau }(Y)\leq {ProbSup}_{\tau }(X)<\sigma . \end{array}$$

By Lemma 2, none of these candidates nor their supersets can be included in the top-*k* result set. Therefore, no further itemsets can improve the current solution, and the search can safely terminate. □

Lemma 3 highlights a fundamental advantage of best-first search: it provides a sound and natural termination condition that is not available under depth-first traversal strategies (Table 4).

**Table 4:** Summary of notation.

Symbol	Description
$J=\{x_{1},\ldots ,x_{m}\}$	Item universe: set of all distinct items in 𝒟; |*J*| = *m* is the number of distinct items (item-universe size)
*X*, *Y*, *Z*	Itemsets (X⊆J)
|*X*|	Cardinality of itemset *X*
$𝒟=\{T_{1},\ldots ,T_{N}\}$	Uncertain transaction database
*N*	Number of transactions, i.e., database size (N=|𝒟|)
$P(x,T_{i})$	Existence probability of item *x* in transaction $T_{i}$
$p_{i}=P(X\subseteq T_{i})$	Probability that itemset *X* appears in transaction $T_{i}$
Sup(X)	Support random variable of *X* (Poisson-binomial)
$F_{X}(s)$	Frequency function: P(Sup(X)≥s)
τ∈(0,1]	Probability threshold (user-defined)
${ProbSup}_{\tau }(X)$	Probabilistic support of *X* at threshold τ
sup(X)	Shorthand for ${ProbSup}_{\tau }(X)$ when τ is clear
ℱ	Set of all $\left(\tau ,s_{\min}\right)$-frequent itemsets
𝒞	Set of all closed frequent itemsets
${𝒞}_{k}$	Set of top-*k* closed frequent itemsets (the algorithm output; $|{𝒞}_{k}|=k$)
*k*	Desired number of top patterns (user-defined)
θ(ℋ)	Dynamic threshold derived from result heap ℋ
σ	Shorthand for θ(ℋ) when context is clear
ℋ	Result min-heap of size *k* (Top-*k* result set)
𝒬	Priority queue of candidates (max-heap)
*V*(*i*)	Probabilistic tidset of item *i*
tidset(X)	Probabilistic tidset of itemset *X*
$m_{X}=|tidset(X)|$	Tidset cardinality of *X*: number of transactions containing *X* (note $m_{X}\leq N$)
${\bar{m}}_{X},\;{\bar{m}}_{2}$	Average tidset cardinality of processed candidates and of cached 2-itemsets
$\bar{M}$	Average transaction width (mean number of items per transaction)
$C_{proc}$	Number of candidate itemsets extracted from 𝒬 and processed during mining (distinct from ${𝒞}_{k}$; typically $C_{proc}\gg k$)
${ℱ}_{1}$	Sorted list of all 1-itemsets from Phase 1
≺	Total order on item universe *J*
$g_{j}(z)$	Generating function polynomial for transaction $T_{j}$
*G*(*z*)	Probability generating function for itemset *X*
*D*[*s*]	Support distribution entry: $P(S_{X}=s)$

### Proposed methods

This section presents TUFCI, a best-first search algorithm for mining top-*k* closed frequent itemsets from uncertain databases. We first describe the overall framework, then detail the data structures, support computation via generating functions, pruning strategies, and the novel support-ordered closure verification technique. Finally, we present the complete algorithm with theoretical analysis.

### Overview of the TUFCI framework

TUFCI addresses the limitations of depth-first search (DFS) approaches by exploring candidates in descending order of probabilistic support using a priority queue. This best-first strategy offers three key advantages over DFS-based methods:

1. **Early discovery of high-support patterns:** By always processing the candidate with highest support first, TUFCI discovers strong patterns early, enabling rapid threshold elevation.
2. **Efficient closure verification:** When checking whether an itemset is closed, TUFCI examines supersets in descending support order, detecting closure violations early and avoiding unnecessary computations.
3. **Safe early termination:** Once *k* closed itemsets have been found and the best remaining candidate has support below the current threshold, TUFCI can safely terminate without exploring the remaining search space.

**Fig 1:**
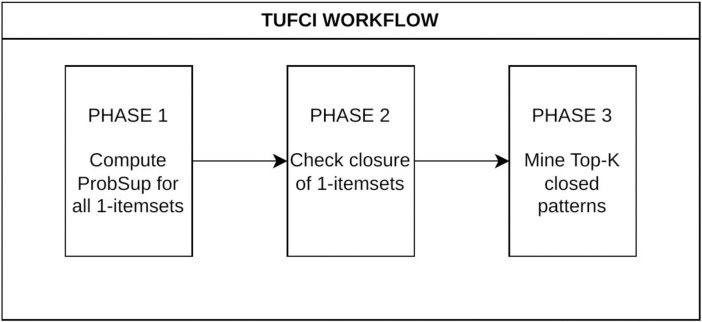
TUFCI framework architecture. The algorithm operates in three phases: (1) computing frequent 1-itemsets from the vertical database, (2) initializing data structures and seeding the priority queue, and (3) best-first mining with support-ordered closure verification. The priority queue ensures candidates are processed in descending support order, enabling early termination when the best candidate falls below the dynamic threshold.

Fig 1 illustrates the complete architecture, highlighting the interaction between the vertical database, priority queue, and the closure verification module.

Algorithm 1 outlines the high-level execution flow of the framework.

**Algorithm 1 TUFCI Framework Overview**

**Require:** Uncertain database 𝒟, parameter *k*

**Ensure:** Top-*k* closed frequent itemsets ℋ

1:  **Phase 1: Preprocessing**

2:  Scan 𝒟 to compute vertical database; compute ${ProbSup}_{\tau }(i)$ for all 1-itemsets

3:  Sort all 1-itemsets by support descending ${ℱ}_{1}$

4:  **Phase 2: Initialization**

5:  Initialize candidate structure 𝒬 and result set ℋ

6:  Seed 𝒬 with frequent 1-itemsets

7:  Initialize dynamic threshold σ←0

8:  **Phase 3: Mining**

9:  **while** 𝒬 is not empty **do**

10:   X←ExtractBest(𝒬)

11:   **if** support(X)<σ **then break**

12:   Process *X* and generate extensions

13:  **end while**

14:  **return** ℋ

As shown in Algorithm 1, the framework operates in three distinct phases:

**Phase 1: Preprocessing (Lines 1–3).** The algorithm scans the uncertain database once to build the vertical representation and computes ${ProbSup}_{\tau }(\{i\})$ for every item *i* in the item universe *J*. No items are filtered at this stage because, in top-*k* mining, the dynamic threshold θ(ℋ) starts at zero and only rises as the result heap fills (see detailed Phase 1 in Section “The TUFCI Algorithm” and Algorithm 4). Items registered here are sorted by descending probabilistic support to produce ${ℱ}_{1}$.

**Phase 2: Initialization (Lines 4–7).** Data structures are initialized. The result heap ℋ maintains the current top-*k* patterns, while the Priority Queue 𝒬 orders candidates. The queue is seeded with frequent 1-itemsets from ${ℱ}_{1}$, setting the stage for the traversal.

**Phase 3: Best-First Mining (Lines 8–14).** This is the core iterative process. In each step, TUFCI extracts the highest-support candidate *X*. Crucially, if *support*(*X*) is lower than the current dynamic threshold σ, the algorithm terminates immediately (Line 11), as no remaining candidate can qualify. Otherwise, closure is verified, results are updated, and valid extensions are generated for future processing.

### Core mining components

The TUFCI algorithm is built upon three core components that enable best-first traversal of the itemset search space: a candidate priority ordering, a dynamic pruning threshold, and a probabilistic vertical data representation. These components are specifically designed to address limitations inherent to depth-first traversal, where candidates are explored according to a fixed enumeration order that is independent of their probabilistic support.

### Candidate priority ordering

Depth-first search expands itemsets according to a predefined lexicographic order, which does not reflect their probabilistic relevance to the top-k objective. As a consequence, itemsets with high probabilistic support may be discovered late in the traversal, delaying threshold updates and weakening pruning effectiveness. In contrast, best-first traversal requires a strict ordering that prioritizes candidates with larger support values.

**Definition 13** (Candidate Priority Order). *For two candidate itemsets X and Y,* X≻Y *(X has higher priority than Y) if:*

1.
support(X)>support(Y) *; or*
2. support(X)=support(Y) *and* $F_{X}(sup(X))>F_{Y}(sup(Y))$*; or*
3. support(X)=support(Y), $F_{X}(sup(X))=F_{Y}(sup(Y))$*, and |X| < |Y|.*

#### Tie-breaking rationale.

When two candidates share the same support value, the priority queue resolves ties by (i) preferring the higher tail-probability value $F_{X}(sup(X))$, then (ii) preferring smaller itemsets — matching the order in Definition 13.

The probability-descending rule (rule 2) is applied first among equal-support candidates: a higher $F_{X}(sup(X))$ value indicates the itemset is more confidently at its support level, making it a more reliable candidate for the result heap. The size-ascending rule (rule 3) then breaks remaining ties: a smaller itemset *X* admits more canonical extensions than a larger *Y* with the same support and probability (since |J⧵X|>|J⧵Y|), causing θ(ℋ) to rise sooner and enabling more aggressive pruning.

#### Empirical sensitivity.

To confirm that the tie-breaking policy does not materially affect our conclusions, we ran a supplementary experiment on the Chess dataset (*k* = 30) with three policies: (a) our default (size-ascending, probability-descending), (b) size-descending, probability-ascending, and (c) random. All three policies produced identical Top-*k* result sets (correctness is guaranteed regardless of tie-breaking). Runtime varied by less than 3% across policies, confirming that the primary performance driver is the support-descending exploration order, not the tie-breaking rule.

This ordering ensures that the search always expands the node with the highest potential first, which is the necessary condition for the *Safe Early Termination* property.

### Dynamic pruning threshold

Unlike traditional mining where the threshold is static, TUFCI maintains a dynamic threshold σ derived from the current set of top-*k* closed patterns found so far.

**Definition 14** (Dynamic support threshold). *Let* ℋ *denote the Top-k min-heap maintained during mining. The dynamic support threshold is*


$$\begin{array}{c} \theta (ℋ)=\left\{ \begin{array}{cc} 0 & \text{if }|ℋ|<k,\\ \min\{{ProbSup}_{\tau }(X):X\in ℋ\} & \text{if }|ℋ|=k. \end{array}\right. \end{array}$$ (21)

*All pruning conditions in Algorithms 4–6 reference* θ *rather than a fixed minimum support. Since* θ *is monotonically non-decreasing over the course of mining (Proposition 1), pruning becomes progressively more aggressive.*

**Property 1** (Monotonicity of θ) *The dynamic threshold* θ(ℋ) *is monotonically non-decreasing over the course of mining.*

*Proof.* The threshold θ can change only upon insertion into ℋ. While |ℋ|<k, insertions do not change θ (which remains 0). When |ℋ|=k, an insertion occurs only if the new pattern *X* satisfies ${ProbSup}_{\tau }(X)>\theta (ℋ)$, and the evicted pattern is the current minimum of ℋ. The new minimum is at least as large as the old minimum, because the evicted element (the previous minimum) has been replaced by a strictly superior element. Hence θ never decreases. □

The threshold σ is a lower bound on the support of any itemset that may belong to the top-k result set. By the anti-monotonicity of probabilistic support, any candidate *X* such that support(X)<σ and all of its supersets can be pruned. Under best-first traversal, high-support itemsets are examined early, causing σ to increase sooner than under depth-first traversal and strengthening pruning effectiveness.

### Probabilistic tidset

To support the rapid support calculations required by the priority queue, we employ a vertical data representation that encapsulates the uncertainty of item occurrences.

**Definition 15** (Probabilistic Tidset). *For an itemset X, the probabilistic tidset is defined as:*


$$\begin{array}{c} tidset(X)=\{(T_{j},P(X,T_{j})):T_{j}\in 𝒟,P(X,T_{j})>0\} \end{array}$$ (22)

*where* $P(X,T_{j})=\prod_{i\in X}P(i,T_{j})$ *assumes independence between items.*

This representation enables efficient support computation and candidate extension via set intersections.

**Example 4.** *Consider the uncertain database in* Table 2. *The corresponding vertical database maps each item to its probabilistic tidset, as shown in* Table 5.

**Table 5:** Vertical representation of the uncertain database.

Item *i*	tidset({i})
*A*	$\left\{(T_{1},0.9),(T_{2},0.8),(T_{3},0.9),(T_{5},0.7)\right\}$
*B*	$\left\{(T_{1},0.8),(T_{2},0.7),(T_{4},0.8),(T_{5},0.6)\right\}$
*C*	$\left\{(T_{1},0.7),(T_{3},0.8),(T_{4},0.9),(T_{5},0.8)\right\}$
*D*	$\left\{(T_{2},0.6),(T_{3},0.7),(T_{5},0.9)\right\}$
*E*	{(*T*_4_,0.5)}

*As illustrated in* Table 5*, the vertical database V is defined by* V(i)=tidset({i}) *for each item* i∈ℐ.

### Priority queue and result heap

The priority queue 𝒬 maintains candidate itemsets ordered according to the **Candidate Priority Order** (Definition 13). This ordering guarantees that, at each iteration, the candidate with the largest probabilistic support is selected for expansion.

The result set ℋ is implemented as a min-heap of fixed size *k*, ordered by probabilistic support. This structure provides constant-time access to the smallest support value among the current top-*k* patterns. When |ℋ|=k, this minimum value defines the dynamic threshold σ, which is used for pruning and early termination.

### Probabilistic support evaluation

In TUFCI, the support of a candidate itemset *X* is a random variable $S_{X}$ representing the number of transactions in which *X* occurs. Assuming independence of item existence events across and within transactions, each transaction contributes a Bernoulli trial with probability $p_{j}=P(X,T_{j})$, where $P(X,T_{j})=\prod_{i\in X}P(i,T_{j})$. Under this assumption, $S_{X}$ follows a Poisson-binomial distribution.

Let σ=θ(ℋ) denote the current dynamic threshold. To determine whether *X* can enter the top-*k* heap, we compute


$$\begin{array}{c} P(S_{X}\geq \sigma )=\sum_{s=\sigma }^{m_{X}}D_{m_{X}}[s], \end{array}$$

where $D_{m_{X}}[s]=P(S_{X}=s)$.

TUFCI computes the distribution $\{D_{m_{X}}[s]{\}}_{s=0}^{m_{X}}$ using a **Direct Convolution** dynamic programming method. For the tidset probabilities $P=[p_{1},p_{2},\ldots ,p_{m_{X}}]$ (one entry per transaction containing *X*), let $D_{i}[s]$ denote the probability that the support equals *s* after processing the first *i* tidset entries. The recurrence is:


$$\begin{array}{c} D_{i}[s]=D_{i-1}[s]\cdot (1-p_{i})+D_{i-1}[s-1]\cdot p_{i}, \end{array}$$ (23)

with *D*_0_[0]=1 and *D*_0_[*s*]=0 for *s*>0.

Algorithm 2 computes this distribution in $O(m_{X}^{2})$ time, where $m_{X}=|tidset(X)|$ is the number of transactions containing *X* (the tidset size), *not* the total database size *N*. This distinction is important: anti-monotonicity guarantees that $m_{X}$ shrinks as itemsets grow longer, so the quadratic cost is paid mainly for short, high-frequency candidates. In the worst case—a single item appearing in all *N* transactions—$m_{X}=N$ and the cost is *O*(*N*^2^), which would be prohibitive. Three mechanisms bound $m_{X}$ in practice: (1) Strategy P7 prunes candidates whose tidset cardinality falls below the dynamic threshold θ(ℋ); (2) longer itemsets have smaller tidsets by anti-monotonicity; and (3) alternative convolution algorithms (e.g., FFT-based or divide-and-conquer) can reduce the per-candidate cost to $O(m_{X}\log^{2}m_{X})$ when $m_{X}$ is large, though the current implementation uses direct convolution throughout. Exact support values are required to correctly order candidates in the priority queue and to enable effective pruning and early termination under the best-first search strategy.

**Algorithm 2 DirectConvolutionSupport**

**Require:** Tidset probabilities of *X*: $P=[p_{1},p_{2},\ldots ,p_{m_{X}}]$

**Ensure:** Support distribution vector *D* (where $D[s]=P(S_{X}=s)$)

1:  Initialize array *D* of size $m_{X}+1$ with zeros

2:  D[0]←1.0 (Probability of support 0 is initially 1)

3:  **for all** p∈P **do**

4:   **for** *j* from $m_{X}$ down to 1 **do**

5:    D[j]←D[j]·(1−p)+D[j−1]·p

6:   **end for**

7:   D[0]←D[0]·(1−p)

8:  **end for**

9:  **return** *D*

### Support-ordered closure verification

Closure verification determines whether an itemset *X* is closed by checking if any of its immediate supersets have the same probabilistic support. In depth-first traversal, extensions are typically examined in a fixed enumeration order that is independent of their support values, which may result in redundant support computations before a closure violation is detected. TUFCI instead evaluates 1-extensions in non-increasing order of support and terminates the check as soon as no remaining extension can match the support of *X*.

The method relies on the anti-monotonicity of probabilistic support with respect to itemset inclusion and on ordering extensions by their support values.

**Theorem 2** (Support-ordered closure verification). *Let X be an itemset with support* $s_{X}$*, and let*


$$\begin{array}{c} E=\{X\cup \{i_{1}\},X\cup \{i_{2}\},\ldots ,X\cup \{i_{m}\}\} \end{array}$$

*be the set of its 1-extensions sorted in non-increasing order of support. If* $support(X\cup \{i_{j}\})<s_{X}$ *for some index j, then for all* ℓ>j *it holds that*


$$\begin{array}{c} support(X\cup \{i_{\ell }\})<s_{X}. \end{array}$$

*Proof.* Because the extensions are sorted in non-increasing order of support, for any ℓ>j we have


$$\begin{array}{c} support(X\cup \{i_{\ell }\})\leq support(X\cup \{i_{j}\}). \end{array}$$

If $support(X\cup \{i_{j}\})<s_{X}$, then $support(X\cup \{i_{\ell }\})<s_{X}$ for all ℓ>j. Hence, once an extension with support strictly smaller than $s_{X}$ is encountered, no subsequent extension can have support equal to $s_{X}$, and the closure check can terminate. □

**Lemma 4** (Closure completeness under sorted iteration). *Let* $ℱ=(i_{1},i_{2},\ldots ,i_{m})$ *be the frequent items sorted in non-increasing order of singleton support. For candidate X with support* $s_{X}$*, if* ${ProbSup}_{\tau }(\{i_{j}\})<s_{X}$ *for some index j, then for all* ℓ≥j*:*


$$\begin{array}{c} {ProbSup}_{\tau }(X\cup \{i_{\ell }\})\;\leq \;{ProbSup}_{\tau }(\{i_{\ell }\})\;\leq \;{ProbSup}_{\tau }(\{i_{j}\})\;<\;s_{X}. \end{array}$$

*Hence no item at position* ≥j *can produce a closure violation (*${ProbSup}_{\tau }(X\cup \{i\})=s_{X}$*).*

*Proof.* By anti-monotonicity (Lemma 1), ${ProbSup}_{\tau }(X\cup \{i_{\ell }\})\leq {ProbSup}_{\tau }(\{i_{\ell }\})$. Since ℱ is sorted in non-increasing order, ${ProbSup}_{\tau }(\{i_{\ell }\})\leq {ProbSup}_{\tau }(\{i_{j}\})<s_{X}$ for all ℓ≥j. The strict inequality precludes equality with $s_{X}$.

#### Remark on sort stability.

The correctness of the closure-done flag requires only that the ordering is non-increasing—not a stable sort. When multiple items share the same singleton support equal to $s_{X}$, all such items are visited *before* the flag is set, because the flag triggers only on a *strict decrease* below $s_{X}$. Any permutation among items with identical support is therefore harmless.

Algorithm 3 implements the combined closure verification and extension generation procedure. It iterates through frequent items in non-increasing order of singleton support, employing two independent control-flow mechanisms:

1. **P3 termination** (line 8): The loop terminates when sup({i})<θ, because by anti-monotonicity sup(X∪{i})≤sup({i})<θ, so no remaining item can produce a viable extension.
2. **Closure-done flag** (line 11): When sup({i})<sup(X), the flag disables closure checking for subsequent items while the loop *continues* to generate extensions. By Lemma 4, no item beyond this point can produce a closure violation.

These two mechanisms are logically independent: P3 terminates the loop entirely (no further extensions are possible), while the closure-done flag only disables one type of check (closure violations are impossible, but extensions may still be viable).

**Algorithm 3 CheckClosureAndExtend**

**Require:** Itemset *X*, dynamic threshold θ

**Ensure:** (isClosed,extensions)

1: isClosed←true

2: closureDone←false {Flag: all remaining items have sup({i})<sup(X)}

3: extensions←∅

4: **for** each item *i* in ℱ in support-descending order **do**

5:   **if** i∈X **then**

6:    **continue**

7:   **end if**

8:   **if** sup({i})<θ **then**

9:     **break** {P3: anti-monotonicity ⇒ sup(X∪{i})≤sup({i})<θ}

10:   **end if**

11:   **if** ¬closureDone **and** sup({i})<sup(X) **then**

12:   closureDone←true (No further closure violations possible (Lemma 4))

13:  **end if**

14:  needClosure←¬closureDone∧isClosed

15:  needExtend←(i>max(X))

16:  **if** ¬needClosure∧¬needExtend **then**

17:   **continue**

18:  **end if**

19:  Compute sup(X∪{i}) via tidset intersection and generating functions

20:  **if** *needClosure* **and** sup(X∪{i})=sup(X) **then**

21:   isClosed←false

22:  **end if**

23:  **if** *needExtend* **and** sup(X∪{i})≥θ **then**

24: extensions←extensions∪{X∪{i}}

25:  **end if**

26: **end for**

27: **return** (isClosed,extensions)

Processing extensions in support order ensures that if a closure-violating superset exists, it will be encountered before any lower-support extension. This reduces the number of support evaluations required for closure verification. Because best-first traversal maintains a global ordering of candidates by support, it increases the likelihood that high-support extensions are generated and checked earlier than under depth-first traversal, which explores candidates according to a fixed structural order. Consequently, support-ordered closure verification complements the best-first search strategy and contributes to reducing redundant computations in TUFCI.

## Best-first search with early termination

### Rationale for the search strategy

Traditional itemset mining algorithms typically employ Depth-First Search (DFS) to traverse the search space. While DFS uses memory efficiently, it traverses candidates in a predetermined structural order that is independent of their probabilistic support values. As a result, itemsets with high probabilistic support—whose early discovery is critical for elevating the dynamic threshold σ—may be encountered late in the traversal. This delay can keep σ low over much of the search and reduce the effectiveness of pruning.

In contrast, TUFCI adopts a Best-First Search (BestFS) strategy based on the priority queue. By always selecting the candidate with the highest probabilistic support for expansion, the algorithm tends to discover high-support itemsets early. Early updates to the dynamic threshold σ strengthen pruning conditions and reduce exploration of low-support regions of the search space.

### Early termination condition

An important property of the BestFS strategy is that it enables safe early termination of the search based on the current dynamic threshold.

**Lemma 5** (Early termination under best-first search). *In the best-first variant (Algorithm 6), if the candidate X returned by* 𝒬.ExtractMax() *satisfies* ${ProbSup}_{\tau }(X)<\theta (ℋ)$*, then all remaining candidates* Y∈𝒬 *also satisfy* ${ProbSup}_{\tau }(Y)\leq {ProbSup}_{\tau }(X)<\theta (ℋ)$*, and mining may safely terminate.*

*Proof.* The proof rests on three facts, each independently verifiable:

(i) *Support immutability.* Each candidate’s probabilistic support is computed once at creation time and stored as an immutable key. The heap key therefore never changes after insertion; the priority queue is a static-key structure from the perspective of any individual candidate.
(ii) *Max-heap invariant.* The priority queue 𝒬 is implemented as a binary max-heap keyed by probabilistic support. The standard binary heap invariant—a property of the data structure independent of the algorithm’s correctness argument—guarantees that ExtractMax(𝒬) returns the element with the largest key in O(log|𝒬|) time and restores the invariant after extraction. Therefore, after extracting X=ExtractMax(𝒬), every remaining element Y∈𝒬 satisfies ${ProbSup}_{\tau }(Y)\leq {ProbSup}_{\tau }(X)$ as a direct consequence of the heap invariant, not as an assumption.
(iii) *Extension pruning.* Given (ii), if ${ProbSup}_{\tau }(X)<\theta (ℋ)$, then ${ProbSup}_{\tau }(Y)\leq {ProbSup}_{\tau }(X)<\theta (ℋ)$ for every remaining Y∈𝒬. Moreover, for any extension Z⊇Y, anti-monotonicity (Lemma 1) gives ${ProbSup}_{\tau }(Z)\leq {ProbSup}_{\tau }(Y)<\theta (ℋ)$, so no extension of any remaining candidate can enter ℋ. Combined with the monotonicity of θ (Property 1), the current Top-*k* result is final.

*Remark on maintenance cost.* The correctness argument above is independent of the computational cost of maintaining the heap ordering. The cost of each Insert and ExtractMax operation is O(log|𝒬|), and the total overhead across the entire mining run is accounted for in the Phase 3 time complexity bound (Section). Crucially, this cost does not affect the validity of the termination condition: once X is extracted and found to satisfy ${ProbSup}_{\tau }(X)<\theta (ℋ)$, termination is correct regardless of how many heap operations were performed to reach that point.

**Fig 2:**
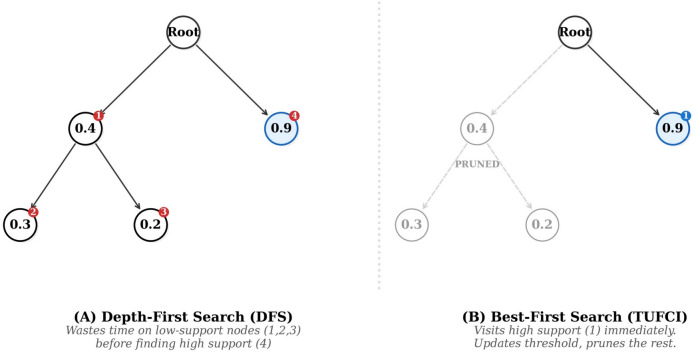
Traversal order comparison. **(A)** Under DFS, exploration proceeds according to structural order. **(B)** Under best-first search, candidates with higher support are visited first, which can lead to earlier threshold updates and pruning.

Fig 2 illustrates this difference in traversal behavior. In the DFS case (A), exploration may proceed deep into branches with low support before reaching high-support itemsets. In the Best-First Search case (B), the highest-support itemsets are visited first, and σ can be updated early, enabling the remaining low-support candidates to be pruned without further expansion.

### The TUFCI algorithm

The TUFCI framework is architected into three distinct phases to efficiently handle the complexity of uncertain data mining. By decoupling preprocessing, initialization, and the core mining loop, the algorithm maximizes modularity and allows for specific optimizations at each stage.

### Phase 1: Preprocessing and singleton support computation

The first phase (Algorithm 4) performs a single linear scan of the database to construct the vertical representation *V*: for each item *i*, the tidset $V(i)=\{(T_{j},P(i,T_{j})):i\in T_{j}\}$ is built. This scan registers every item into the item universe *J*, including items that appear only in late transactions. Phase 1 then iterates over *J* (not over transactions) and computes ${ProbSup}_{\tau }(\{i\})$ for each i∈J using *V*(*i*). No item can be missed regardless of its position in the database. The per-item computation cost is *O*(|*V*(*i*)|^2^) via dynamic-programming convolution over only the |*V*(*i*)| transactions containing item *i*.

Crucially, Phase 1 does *not* filter items by a minimum support threshold, because in top-*k* mining the dynamic threshold θ(ℋ) starts at zero and is derived from the Top-*k* heap state (Definition 14). Filtering occurs in Phase 2 as the heap fills.

#### Addressing the late-appearing items concern.

A natural question arises: does TUFCI’s single-pass preprocessing miss items that appear only in late transactions? The answer is *no*, by construction. The vertical database build (Algorithm 4, Line 1) scans *all N* transactions sequentially, registering every observed item into item universe *J* regardless of transaction index. Consider a concrete example:

**Example (Late-appearing item):** Suppose a database contains 5 transactions: *T*_1_ = {*A*, *B*}, *T*_2_ = {*B*, *C*}, *T*_3_ = {*A*, *C*}, *T*_4_ = {*B*, *D*}, *T*_5_ = {*E*}. Item *E* appears only in the final transaction. During the vertical scan:

1. Transactions $T_{1}-T_{4}$ are processed, registering *A*, *B*, *C*, *D* into *J* and populating their tidsets.
2. Transaction *T*_5_ is processed, registering *E* into *J* and creating $V(E)=\{(T_{5},P(E,T_{5}))\}$.
3. After the scan completes, *J* = {*A*, *B*, *C*, *D*, *E*}, and Phase 1 computes ProbSup({E}) using its tidset like any other item.

Mathematically, the vertical database construction guarantees that for *every* item *i* occurring in *at least one* transaction $T_{j}\in 𝒟$, we have i∈J after the scan completes. The subsequent support computation (Algorithm 4, Lines 3–7) iterates over *J*, not over a pre-filtered set based on position. Therefore, late-appearing items are *never* excluded by position bias; they are treated identically to early-appearing items. The only reason an item might be filtered later (Phase 2 or Phase 3) is if its probabilistic support falls below the dynamically rising threshold θ(ℋ)—a correctness condition, not a positional artifact.

#### Preprocessing step.

Before mining begins, the algorithm performs a single linear scan of the entire database 𝒟 to construct the vertical representation *V*. During this scan, every item encountered is registered in the item universe *J*, and for each item *i*, the tidset $V(i)=\{(T_{j},P(i,T_{j})):i\in T_{j}\}$ is assembled. This preprocessing step has time complexity $O(N\cdot \bar{M})$, where N=|𝒟| is the number of transactions and $\bar{M}$ is the average transaction width. Crucially, no item is missed regardless of its position in the database—an item appearing only in the final transaction is registered and its tidset is constructed during this single pass, as demonstrated in the example above.

**Algorithm 4 Phase 1: Preprocessing**

**Require:** Uncertain database 𝒟, probability threshold τ

**Ensure:** Sorted list of all 1-itemsets ${ℱ}_{1}$, vertical database *V*

1: V←BuildVerticalDatabase(𝒟) {Single linear scan; registers all items into item universe *J*}

2: ${ℱ}_{1}\leftarrow$ empty list

3: **for all** item i∈J **do**

4:  ${tidset}_{i}\leftarrow V.\mathrm{GetTidset}(i)$

5:  **if** ${tidset}_{i}$ is empty **then**

6:   **continue**

7:  **end if**

8:  $\left({sup}_{i},{prob}_{i})\leftarrow \mathrm{ComputeProbabilisticSupport}({tidset}_{i},\tau \right)$

9:  ${ℱ}_{1}.\mathrm{Add}(\{i\},{sup}_{i},{prob}_{i},{tidset}_{i})$

10: **end for**

11: Sort ${ℱ}_{1}$ by support descending

12: **return** $\left({ℱ}_{1},V\right)$

### Phase 2: Initialization

In the second phase (Algorithm 5), we initialize the core data structures: the **Top-*k* Heap** (ℋ) to store the current best closed itemsets, and the **Max-Priority Queue** (𝒬) to manage the search frontier.

The Priority Queue 𝒬 is seeded with the frequent 1-itemsets identified in Phase 1 (line 10 of Algorithm 5); these are the initial candidates for expansion. In addition, Phase 2 pre-computes and caches 2-itemset supports (line 11) in a separate hash table ${𝒞}_{2}$ that supports the P4 upper-bound pruning strategy; the 2-itemset cache is an auxiliary lookup structure and its entries are *not* inserted into 𝒬. A preliminary closure check is performed on each 1-itemset: if it is subsumed by a superset with equal support, it is not added to ℋ as a closed candidate, though it remains available in 𝒬 for extension generation.

**Algorithm 5 Phase 2: Initialization**

**Require:** Sorted frequent 1-itemsets ${ℱ}_{1}$, integer *k*, threshold τ

**Ensure:** Priority Queue 𝒬, Result Heap ℋ, 2-itemset cache ${𝒞}_{2}$

1: ℋ←MinHeap(k) {θ(ℋ)=0 initially}

2: 𝒬←MaxPriorityQueue()

3: ${𝒞}_{2}\leftarrow \mathrm{HashMap}()$ (Auxiliary 2-itemset support cache for P4)

4: **for all** $(\{i\},{sup}_{i},{prob}_{i},{tidset}_{i})\in {ℱ}_{1}$ **do**

5:  **if** ℋ is full **and** ${sup}_{i}<\theta (ℋ)$ **then**

6:   **break** {P1: remaining singletons cannot enter ℋ)}

7:  **end if**

8:  $isClosed\leftarrow \mathrm{CheckClosure1Itemset}(\{i\},{ℱ}_{1},\theta (ℋ))$

9:  **if** *isClosed* **then**

10:   $ℋ.\mathrm{Insert}(\{i\},{sup}_{i},{prob}_{i})$ {θ(ℋ) may increase}

11:  **end if**

12:  $𝒬.\mathrm{Insert}(\{i\},{sup}_{i},{prob}_{i},{tidset}_{i})$ {Seed 𝒬 with 1-itemset for later expansion}

13: **end for**

14: Pre-compute and store sup({i,j}) in ${𝒞}_{2}$ for all pairs $i,j\in {ℱ}_{1}$ with ${sup}_{i},{sup}_{j}\geq \theta (ℋ)$

15: **return** $\left(𝒬,ℋ,{𝒞}_{2}\right)$

### Phase 3: Best-first mining process

The core mining loop is presented in Algorithm 6. This phase iteratively extracts the candidate with the highest support from 𝒬 and performs three critical operations:

1. **Early Termination:** If the support of the extracted candidate is less than the current dynamic threshold σ, the algorithm terminates immediately. Since 𝒬 is sorted, no remaining candidate can possibly qualify for the top-*k* set (Lemma 5).
2. **Expansion & Closure:** The candidate is expanded to generate supersets. We simultaneously check if the candidate is closed.
3. **Result Update:** If closed, it is added to ℋ. If ℋ becomes full, σ is raised to the support of the *k*-th best pattern, effectively pruning the remaining search space.

**Algorithm 6 Phase 3: Best-First Mining**

**Require:** Queue 𝒬, Heap ℋ, threshold τ

**Ensure:** Final Top-*k* closed frequent itemsets ℋ

1: **while** 𝒬 is not empty **do**

2:  X←𝒬.ExtractMax()

3:  **if** sup(X)<θ(ℋ) **then**

4:   **break** (P2: early termination (Lemma 5))

5:  **end if**

6:  (isClosed,extensions)←CheckClosureAndExtend(X,θ(ℋ))

7:  **if** *isClosed* **then**

7:   ℋ.Insert(X) {θ(ℋ) may increase}

8:  **end if**

9:  **for all** Y∈extensions **do**

10:   **if** sup(Y)≥θ(ℋ) **then**

11:    𝒬.Insert(Y)

12:   **end if**

13:  **end for**

14: **end while**

15: **return** ℋ

### Execution walkthrough with example

To clarify the interaction between these phases, consider the uncertain database from Table 2 with threshold τ=0.7 and *k* = 2.

**Phase 1 (Preprocessing):** Items are evaluated. Suppose the computed probabilistic supports are: *A* = 3, *C* = 3, *B* = 2, *D* = 2. Sorted ${ℱ}_{1}=\langle A,C,B,D\rangle$.

**Phase 2 (Initialization).** Priority Queue 𝒬 is seeded with the frequent 1-itemsets.


$$\begin{array}{c} 𝒬=[(A,3),(C,3),(B,2),(D,2)] \end{array}$$ (24)

Result heap ℋ is empty; σ=0

**Phase 3 (Mining).**

- **Iteration 1:** Extract *A* (support 3). Check extensions (*AB*, *AC*). Assume extensions have support 2. Since *sup*(*A*)> *sup*(*extensions*), *A* is **Closed**.
  - Add *A* to ℋ. Top-*k*: {*A*}.
  - Add *AB*, *AC* to 𝒬.
- **Iteration 2:** Extract *C* (support 3). Assume *C* is closed.
  - Add *C* to ℋ. Top-*k*: {*A*, *C*}.
  - **Update** σ: ℋ is full (*k* = 2). Minimum support in ℋ is 3. New σ=3.
- **Iteration 3:** Extract next best candidate (e.g., *B* or *AB*, which have support 2).
- **Termination Check:** Current candidate support (2) <σ (3).
- **Action:** Algorithm breaks immediately.

This demonstrates how TUFCI avoids processing low-support patterns (B,D,…) entirely once the Top-*k* buffer is filled with high-quality patterns.

### Pruning strategies

To address the exponential search space inherent in uncertain databases, TUFCI integrates seven distinct pruning strategies (P1–P7) organized into four semantic groups. We note that strategies P2 (dynamic-threshold pruning), P3 (item-level filtering), and P5 (upper-bound filtering, which applies the P4 test as a pre-convolution filter) are established techniques in deterministic and utility-based pattern mining [6,13,14], while P4 (subset-based upper bound) generalises the pairwise upper bound used in top-*k* HUIM [13,14] to the probabilistic-support setting. The novel contributions of TUFCI’s pruning framework are P1 (safe early termination unique to best-first traversal under closure verification), P6–P7 (tidset-cardinality bounds for skipping convolution before its $O(m_{X}^{2})$ cost is paid), and—most importantly—the integration of all seven strategies under support-ordered exploration, which makes their joint effect super-additive (Exp 2).

- **G1 (Frontier Termination)**: P1 (Early Termination), P2 (Dynamic Threshold Pruning) — control global search space by raising and enforcing the dynamic threshold θ(ℋ).
- **G2 (Item Admissibility)**: P3 (Item-Level Filtering) — preprocesses the database to remove infrequent items.
- **G3 (Upper Bound Pruning)**: P4 (Subset-Based Upper Bound), P5 (Upper Bound Filtering) — exploit anti-monotonicity and cached 2-itemset supports to avoid expensive convolution.
- **G4 (Tidset Optimization)**: P6 (Tidset Intersection Look-ahead), P7 (Tidset-Based Early Closure Detection) — leverage tidset cardinality bounds to skip convolution when possible.

These strategies are applied hierarchically, prioritizing low-cost checks (*O*(1) or *O*(*N*)) to eliminate unpromising candidates before invoking the computationally expensive convolution algorithm ($O(m_{X}^{2})$, where $m_{X}=|tidset(X)|$).

### Strategy 1: Early termination (P1)

This strategy, unique to the best-first approach, enables safe termination of the entire search once the priority queue head falls below the dynamic threshold. Experimental results (Exp 4) show it is the most impactful single pruning component, particularly on dense datasets where the threshold rises rapidly after discovering high-support patterns early.


$$\begin{array}{c} \text{If }𝒬.peek().support<\theta (ℋ)\Rightarrow \text{Terminate global search} \end{array}$$ (25)

### Strategy 2: Dynamic threshold pruning (P2)

As the mining progresses and *k* patterns are discovered, the internal threshold σ rises. By Lemma 2, any candidate *X* with ProbSup(X)<σ and all its supersets can be safely pruned, since no descendant can reach the threshold.

### Strategy 3: Item-level filtering (P3)

In Phase 1, once the dynamic threshold θ(ℋ) has been established from the initial closed 1-itemsets, any item *i* with ProbSup({i})<θ(ℋ) can be permanently removed, since by anti-monotonicity no itemset containing *i* can exceed θ(ℋ).


$$\begin{array}{c} {𝒟}^{\prime }=𝒟⧵\{i\in ℐ:ProbSup(\{i\})<\theta (ℋ)\} \end{array}$$ (26)

This strategy effectively reduces the width of the lattice and provides consistent performance gains across dense datasets.

### Strategy 4: Subset-based upper bound (P4)

When extending candidate *X* with item *i*, the basic anti-monotonicity bound gives sup(X∪{i})≤min(sup(X),sup({i})). Strategy P4 tightens this bound by exploiting cached 2-itemset supports from Phase 2.

**Property 2** (Subset upper bound) *For any itemset X and item* i∉X*:*


$$\begin{array}{c} sup(X\cup \{i\})\;\leq \;\min_{y\in X}\;sup(\{y,i\}). \end{array}$$ (27)

*Proof.* For each y∈X, {y,i}⊆X∪{i}, so sup(X∪{i})≤sup({y,i}) by Lemma 1. Taking the minimum over all y∈X yields the bound. □

P4 iterates over items y∈X, looks up each canonical 2-itemset {min(y,i),max(y,i)} in the cache, and tightens the upper bound. Each lookup is *O*(1) expected time (hash-map access). If the tightened bound falls below θ(ℋ), the extension is pruned, saving one $O(m_{X\cup \{i\}}^{2})$ convolution.

#### Example.

Let *X* = {*A*, *B*} with sup(X)=45 and extension item *C* with sup({C})=50. The basic bound is min(45,50)=45. Phase 2 cached sup({A,C})=38 and sup({B,C})=41. After P4: ub=min(45,38,41)=38. If θ(ℋ)=40, then 38 < 40, so extension {*A*, *B*, *C*} is pruned without computing its actual support.

### Strategy 5: Upper bound filtering (P5)

After P4 computes the tightened upper bound *ub* for extension X∪{i}, P5 compares it against the current threshold:


$$\begin{array}{c} \text{If }ub<\theta (ℋ)\;\Rightarrow \;\text{skip extension }X\cup \{i\}. \end{array}$$ (28)

This is an *O*(1) check that prevents the algorithm from entering the expensive tidset-intersection and convolution stages for extensions that cannot enter the Top-*k* heap.

### Strategy 6: Tidset intersection look-ahead (P6)

When expanding a candidate *X* with item *i*, we compute the size of the intersection of their tidsets |tidset(X)∩tidset({i})| before generating the new node.


$$\begin{array}{c} \text{If }|tidset(X)\cap tidset(\{i\})|<\theta (ℋ)\Rightarrow \text{Prune extension }X\cup \{i\} \end{array}$$ (29)

While highly effective for sparse data, this strategy is applied conditionally, as the intersection overhead can outweigh the benefits in very dense datasets.

### Strategy 7: Tidset-based early closure detection (P7)

After computing the tidset intersection 𝒱(X∪{i})=𝒱(X)∩𝒱({i}), the cardinality |𝒱(X∪{i})| is available before the expensive convolution step.

**Property 3** (Tidset cardinality bound) *For any itemset X,* ${ProbSup}_{\tau }(X)\leq |𝒱(X)|$*.*

*Proof.* $Sup(X)=\sum_{t\in 𝒱(X)}B_{t}^{X}$ is a sum of |𝒱(X)| Bernoulli variables, so Sup(X)≤|𝒱(X)| almost surely. For any s>|𝒱(X)|, Pr(Sup(X)≥s)=0<τ. □

P7 exploits this bound in two logically distinct ways, each targeting a different expensive operation:

1. **Skip convolution entirely (extension pruning):** If |𝒱(X∪{i})|<θ(ℋ), then by Property 3, sup(X∪{i})≤|𝒱(X∪{i})|<θ(ℋ), so the extension can never enter the heap. If no closure check is needed for this item (either *closureDone* is already set, or i≤max(X) making it non-canonical), the $O(m_{X\cup \{i\}}^{2})$ convolution is skipped entirely.
2. **Clear closure-check flag (closure pruning):** If |𝒱(X∪{i})|<sup(X), then sup(X∪{i})≤|𝒱(X∪{i})|<sup(X), so a closure violation (sup(X∪{i})=sup(X)) is impossible. The closure-check flag is cleared, saving the equality comparison. If the extension is also not needed (i≤max(X)), convolution is skipped; otherwise it proceeds only to compute the extension support value.

#### Example 1 (Branch 2 — closure pruning).

Let *X* = {*A*, *B*} with sup(X)=20 and θ(ℋ)=15. For item *C* with sup({C})=25, we compute |𝒱({A,B,C})|=17. Since 15 ≤ 17 (extension cannot be pruned by Branch 1), but 17<20=sup(X) (Branch 2 fires), Property 3 guarantees sup({A,B,C})≤17<20, so no closure violation is possible and the closure-check flag is cleared. If C>max(X) (canonical extension), convolution still runs to obtain the support value for heap insertion; the savings are in avoiding the closure equality test.

#### Example 2 (Branch 1 — skip convolution entirely).

Using the same *X* = {*A*, *B*} with sup(X)=20 and θ(ℋ)=15. Now consider item *E* with sup({E})=8. We compute |𝒱({A,B,E})|=12. Since 12<15=θ(ℋ) (Branch 1 fires), the extension {*A*,*B*,*E*} can never reach the threshold regardless of its exact support. Both the closure check *and* the *O*(*m*^2^) convolution are skipped entirely. This is the most aggressive saving: one tidset intersection ($O(m_{X})$) replaces what would otherwise be a full convolution ($O(m_{X}^{2})$).

**Theorem 3** (Pruning Correctness). *The pruning strategies P1–P7 do not eliminate any true top-k closed frequent itemset.*

*Proof.* We treat each strategy in turn. Throughout, let σ=θ(ℋ) denote the dynamic threshold.

*P1 (early termination).* When the head of 𝒬 extracted by ExtractMax has sup(X)<σ, Lemma 3 guarantees that all remaining candidates and their supersets have support <σ and thus cannot enter ℋ.

*P2 (dynamic threshold pruning).* If sup(X)<σ, Lemma 2 ensures that no superset of *X* has support ≥σ. Pruning *X* and its supersets is therefore safe.

*P3 (item-level filtering).* An item *i* with sup({i})<σ cannot appear in any itemset with support ≥σ by Lemma 1, so removing *i* from the item universe *J* cannot eliminate any top-*k* candidate.

*P4 (subset-based upper bound).* For extension X∪{i}, Property 2 states $sup(X\cup \{i\})\leq \min_{y\in X}sup(\{y,i\})$. If this upper bound is <σ, then sup(X∪{i})<σ, so X∪{i} cannot enter ℋ and may be pruned.

*P5 (upper bound filtering).* P5 simply applies the test ub<σ to the upper bound *ub* computed by P4 (or any tighter upper bound). Correctness follows from P4.

*P6 (tidset intersection look-ahead).* For any itemset *Z*, Property 3 gives sup(Z)≤|𝒱(Z)|. If |𝒱(X∪{i})|<σ, then sup(X∪{i})<σ, so the extension cannot enter ℋ.

*P7 (tidset-based early closure detection), Branch 1 (skip convolution entirely)* If |𝒱(X∪{i})|<σ, by Property 3 the extension fails the threshold; skipping convolution is equivalent to a P6-style prune.

*P7, Branch 2 (clear closure-check flag).* If |𝒱(X∪{i})|<sup(X), by Property 3 we have sup(X∪{i})≤|𝒱(X∪{i})|<sup(X), which precludes a closure violation sup(X∪{i})=sup(X). Clearing the closure-check flag therefore omits only equality tests that are already guaranteed to fail; no genuine closure violation is missed.

Combining the per-strategy arguments, every pruned itemset is either provably outside the top-*k* frontier (P1–P6 and P7 Branch 1) or is irrelevant to closure determination (P7 Branch 2). Therefore no true top-*k* closed frequent itemset is eliminated (Table 6).

**Table 6:** Summary of the seven pruning strategies. *Type* indicates whether a strategy is a *standard* technique from prior pattern-mining work, an *adapted* bound transferred to the probabilistic-support setting, or *new* to this work; the classification follows the discussion at the start of this subsection. Here θ(ℋ) is the dynamic threshold, *ub* the subset-based upper bound, and 𝒱(·)=tidset(·).

Strategy	Group	Trigger condition ⇒ action (purpose)	Type
P1 (Early termination)	G1	𝒬.peek().sup<θ(ℋ)⇒ stop the entire search; no remaining candidate or its supersets can enter ℋ	New
P2 (Dynamic threshold)	G1	sup(X)<θ(ℋ)⇒ prune *X* and all its supersets	Standard
P3 (Item-level filtering)	G2	sup({i})<θ(ℋ)⇒ remove item *i* from the lattice, reducing its width	Standard
P4 (Subset-based upper bound)	G3	$\min_{y\in X}sup(\{y,i\})<\theta (ℋ)\Rightarrow$ prune X∪{i}; tightens the bound using cached 2-itemset supports, saving one $O(m_{X}^{2})$ convolution	Adapted
P5 (Upper-bound filtering)	G3	ub<θ(ℋ)⇒ skip X∪{i} before the tidset-intersection and convolution stages (applies the P4 bound)	Standard
P6 (Tidset look-ahead)	G4	|tidset(X)∩tidset({i})|<θ(ℋ)⇒ prune X∪{i} before node creation	New
P7 (Tidset-based early closure)	G4	|𝒱(X∪{i})|<θ(ℋ)⇒ skip convolution entirely; or |𝒱(X∪{i})|<sup(X)⇒ clear the closure-check flag	New

### Complexity analysis

This subsection provides formal time and space complexity bounds for TUFCI. Throughout, $m_{X}=|tidset(X)|$ denotes tidset cardinality, not database size *N*. We emphasise at the outset that TUFCI does *not* improve the worst-case asymptotic complexity of top-*k* closed mining: without pruning it processes the same *O*(2^|*J*|^) candidates as a depth-first baseline (see “Worst-Case Bound” below). Its contribution is to *practical*, average-case efficiency—reducing the number of candidates actually processed, $C_{proc}$, through support-ordered exploration and safe early termination.

### Time complexity

#### Phase 1: Preprocessing.

The single linear database scan constructs the vertical representation in $O(N\cdot \bar{M})$ time, where N=|𝒟| is the number of transactions and $\bar{M}$ is the average transaction width. Computing probabilistic support for each 1-itemset requires polynomial convolution over its tidset: for item *i* with tidset cardinality $m_{i}=|V(i)|$, the direct convolution method (Section) costs $O(m_{i}^{2})$. Summing over all items in item universe *J*:


$$\begin{array}{c} T_{\text{Phase1}}=O\left(N\cdot \bar{M}+\sum_{i\in J}m_{i}^{2}\right). \end{array}$$ (30)

In the worst case where every item appears in all transactions ($m_{i}=N$ for all *i*), this becomes $O(N\cdot \bar{M}+|J|\cdot N^{2})$.

#### Phase 2: Initialization.

Inserting $\left|{ℱ}_{1}\right|$ frequent 1-itemsets into the priority queue requires $O(|{ℱ}_{1}|\log k)$ time (min-heap operations for the result heap ℋ). Closure checks for 1-itemsets are $O(|{ℱ}_{1}{|}^{2})$ in the worst case (checking all pairs). Generating and caching 2-itemsets involves $O(|{ℱ}_{1}{|}^{2})$ pairs, each requiring tidset intersection (*O*(*N*)) and convolution ($O(m_{ij}^{2})$ where $m_{ij}$ is the intersection size). Total:


$$\begin{array}{c} T_{\text{Phase2}}=O\left(|{ℱ}_{1}{|}^{2}\cdot (N+{\bar{m}}_{2}^{2})\right), \end{array}$$ (31)

where ${\bar{m}}_{2}$ is the average tidset size of 2-itemsets.

#### Phase 3: Best-First Mining.

Let $C_{proc}$ denote the number of candidates extracted from the priority queue during mining (a dataset-dependent value bounded by the search space size). For each candidate *X* with tidset cardinality $m_{X}$:

- **Priority queue operations**: O(log|𝒬|) per pop and push. The max queue size |𝒬| is bounded by the width of the itemset lattice, typically *O*(|*J*|^2^) after pruning. Total PQ cost: $O(C_{proc}\log|𝒬|)$.
- **Extension generation**: For each candidate *X*, generating extensions involves iterating over *O*(|*J*|) candidate items (reduced by P3 filtering). For each extension item *i*:
  - Tidset intersection: $O(m_{X}+m_{i})=O(N)$ worst-case
  - Probabilistic support convolution: $O(m_{X\cup \{i\}}^{2})$, where $m_{X\cup \{i\}}\leq \min(m_{X},m_{i})$
- **Closure checking**: For each candidate entering the result heap, closure verification examines *O*(|*J*|) potential supersets (Lemma 4). Each closure check may require support comparison, which is *O*(1) if support is cached or $O(m_{superset}^{2})$ if recomputation is needed. With caching (our implementation), closure cost is *O*(|*J*|) per candidate.

Combining these components and noting that pruning strategies (P1–P7) reduce $C_{proc}$ significantly:


$$\begin{array}{c} T_{\text{Phase3}}=O\left(C_{proc}\cdot \left(\log|𝒬|+|J|\cdot {\bar{m}}_{X}^{2}+|J|\right)\right), \end{array}$$ (32)

where ${\bar{m}}_{X}$ is the average tidset cardinality of candidates processed.

This expression makes the best-first trade-off explicit. The $C_{proc}\cdot O(\log|𝒬|)$ term is the priority-queue maintenance overhead that a stack-based DFS (with *O*(1) push/pop) does not incur. TUFCI pays it deliberately in order to keep $C_{proc}$ small: by expanding candidates in descending support order, the threshold θ(ℋ) rises early, so the dominant per-candidate work—the $O(|J|\cdot {\bar{m}}_{X}^{2})$ support convolutions and the *O*(|*J*|) closure checks—is never paid on the many low-support candidates that a DFS would expand before its threshold rises. In short, best-first trades a logarithmic queue cost for a reduction in the number of expensive support and closure computations.

#### Worst-case bound.

In the absolute worst case without pruning, $C_{proc}=O(2^{\left|J\right|})$ (exponential in item-universe size) and ${\bar{m}}_{X}=N$. However, this scenario is unrealistic in practice: pruning strategies (especially P1, P2, P4, P5) reduce $C_{proc}$ by multiple orders of magnitude. Empirically, $C_{proc}$ grows sub-exponentially with *k* (Exp 2 and Exp 3, Fig 4 and Fig 5, show that TUFCI processes 10–100× fewer candidates than naive DFS on dense datasets).

#### Best-case bound.

When top-*k* patterns are discovered early (as in best-first search), the dynamic threshold θ(ℋ) rises rapidly, triggering early termination (P1). In this case, $C_{proc}\approx O(k\cdot |J|)$ (only patterns near the top-*k* frontier are explored), yielding near-linear complexity in *k*:


$$\begin{array}{c} T_{\text{best}}=O\left(k\cdot |J|\cdot {\bar{m}}_{X}^{2}\right). \end{array}$$ (33)

### Space complexity

#### Vertical Database.

Storing the vertical representation requires $O(N\cdot \bar{M})$ space to store all item-transaction-probability triples.

#### Priority Queue.

The max-priority queue 𝒬 stores candidate itemsets with their tidsets. In the worst case, |𝒬| can reach *O*(|*J*|^2^) (all 2-itemsets), but pruning (especially P4, P5) keeps |𝒬| much smaller. Each candidate stores: (1) itemset (average $\bar{\ell }$ items), (2) tidset (average ${\bar{m}}_{X}$ transactions), (3) support value. Total queue space:


$$\begin{array}{c} S_{𝒬}=O\left(|𝒬|\cdot (\bar{\ell }+{\bar{m}}_{X})\right). \end{array}$$ (34)

#### Result Heap.

The min-heap ℋ stores exactly *k* itemsets with their metadata. Space: $O(k\cdot (\bar{\ell }+{\bar{m}}_{X}))$.

#### 2-Itemset Cache.

Phase 2 caches up to $O(|{ℱ}_{1}{|}^{2})$ 2-itemset supports for P4 upper-bound pruning. Each entry stores a pair of items and a support value: $O(|{ℱ}_{1}{|}^{2})$ space.

#### Total Space.

Combining all components:


$$\begin{array}{c} S_{\text{total}}=O\left(N\cdot \bar{M}+|𝒬|\cdot {\bar{m}}_{X}+k\cdot {\bar{m}}_{X}+|{ℱ}_{1}{|}^{2}\right). \end{array}$$ (35)

Typically, $|{ℱ}_{1}|\ll N$ (items are far fewer than transactions), and pruning keeps $|𝒬|\ll 2^{\left|J\right|}$. Experimental results (Exp 7, Fig 9) show that V1 (TUFCI-BestFS with full pruning) maintains peak heap memory comparable to DFS variants, confirming that the priority queue overhead is manageable.

### Summary table

Table 7 summarizes the per-phase and per-operation complexity bounds.

**Table 7:** Time and space complexity summary for TUFCI.

Component	Time Complexity	Space Complexity
Phase 1 (Preprocessing)	$O(N\cdot \bar{M}+\sum_{i}m_{i}^{2})$	$O(N\cdot \bar{M})$
Phase 2 (Initialization)	$O(|{ℱ}_{1}{|}^{2}\cdot {\bar{m}}_{2}^{2})$	$O(|{ℱ}_{1}{|}^{2})$
Phase 3 (Mining)	$O(C_{proc}\cdot |J|\cdot {\bar{m}}_{X}^{2})$	$O(|𝒬|\cdot {\bar{m}}_{X}+k)$
PQ operation (per candidate)	O(log|𝒬|)	—
Convolution (per candidate)	$O(m_{X}^{2})$	—
Closure check (per candidate)	*O*(|*J*|)	—
**Total (typical case)**	$O(k\cdot |J|\cdot {\bar{m}}_{X}^{2})$	$O(N\cdot \bar{M}+|𝒬|\cdot {\bar{m}}_{X}+|{ℱ}_{1}{|}^{2})$

*Note on the summary table* For readability, Table 7 reports only the *dominant* term of each phase; the lower-order terms retained in the per-phase analysis above are omitted here. Specifically, Phase 2 also incurs an $O(|{ℱ}_{1}{|}^{2}N)$ tidset-intersection cost, and Phase 3 also incurs a $C_{proc}\cdot O(\log|𝒬|)$ heap-maintenance term and an additive $C_{proc}\cdot O(|J|)$ term. The “Total (typical case)” row corresponds to the effective-pruning regime and coincides with the best-case bound $T_{best}=O(k\;|J|\;{\bar{m}}_{X}^{2})$ derived below.

#### Notation clarification.

Throughout this paper, when we write $O(m_{X}^{2})$ for convolution complexity, $m_{X}$ denotes the *tidset cardinality* of itemset *X* (i.e., $m_{X}=|tidset(X)|$), **not** the database size *N*. The quadratic term is in the tidset size, which is typically much smaller than *N* due to item infrequency. For example, if itemset *X* appears in only 100 transactions out of *N* = 88,162 (Retail dataset), then $m_{X}=100$ and convolution costs *O*(100^2^) = *O*(10,000) operations, not *O*(*N*^2^).

#### Overall end-to-end complexity and comparison with DFS.

The dominant cost across all three phases is the $O(m_{X}^{2})$ convolution per candidate. The key quantity determining total runtime is therefore $C_{proc}$, the number of candidates actually processed. In the **worst case** (no pruning), both TUFCI and a DFS baseline process *O*(2^|*J*|^) candidates and have identical asymptotic complexity. The algorithmic advantage of TUFCI lies entirely in **reducing** $C_{proc}$ **in practice**: by processing candidates in descending support order, TUFCI fills the result heap ℋ early, raises θ(ℋ) rapidly, and triggers early termination (P1) before the bulk of the search space is reached. A DFS baseline, exploring in enumeration order, may keep θ(ℋ) near zero for a large fraction of its run, deferring pruning and forcing convolution on many low-support candidates that TUFCI never touches.

Concretely, in the **best-case** where the top-*k* patterns are the highest-support singletons and their immediate extensions, TUFCI terminates after processing O(k·|J|) candidates, giving total time $O(k\cdot |J|\cdot {\bar{m}}_{X}^{2})$, which is near-linear in *k*. An equivalent DFS would still explore $\Omega (|J{|}^{2})$ candidates before reaching the same termination condition. Space complexity is $O(N\cdot \bar{M}+|𝒬|\cdot {\bar{m}}_{X})$; the $|𝒬|\cdot {\bar{m}}_{X}$ term represents the priority queue overhead absent in stack-based DFS, but experimental results (Exp 7, Section) show this overhead is bounded well below the vertical database cost $N\cdot \bar{M}$ on all tested datasets.

## Performance evaluation

### Dataset synthesis and uncertainty generation

Since standard benchmark datasets from the SPMF repository are deterministic, we generated uncertain databases using a synthetic injection method consistent with experimental protocols in probabilistic frequent itemset mining literature. The transformation follows the tuple-independent model, where every item occurrence is associated with an independent existence probability.

To ensure realistic data characteristics, we employed a three-stage generation process (Random Seed = 42):

1. **Support-Correlated Probability:** We reflect the real-world observation that frequent items often have higher data quality. The base probability $P_{base}(i)$ for an item *i* is assigned proportional to its normalized frequency *f*(*i*):
  $$\begin{array}{c} P_{base}(i)=P_{min}+(P_{max}-P_{min})\times f(i{)}^{\alpha } \end{array}$$ (36)
  where $P_{min}=0.1$, $P_{max}=1.0$, and the skew exponent α=1.0.
2. **Transaction Length Adaptation:** To simulate sensor noise where larger transactions are more prone to errors, we apply a penalty factor to transactions exceeding the average length $\mu_{len}$:
  $$\begin{array}{c} P_{final}(i,T)=P_{base}(i)\times \left(1-0.5\times \frac{\max(0,|T|-\mu_{len})}{\mu_{len}}\right) \end{array}$$ (37)
3. **Noise Injection:** To test robustness, we injected random noise with probability ρ=0.02, decoupling specific items from their support and assigning them a random low probability in the range $\left[0.05,P_{base}\right]$.

## Experimental setup

### Datasets

We selected four real-world datasets representing diverse mining scenarios to evaluate the framework’s adaptability:

- **Dense datasets (Chess, Mushroom):** Characterized by a small item universe but high item frequency and strong correlations. These datasets generate long patterns and heavily stress the closure verification and probabilistic support computation components.
- **Sparse datasets (Retail, Liquor):** Characterized by large item universes and variable transaction lengths. These datasets test the algorithm’s scalability and the effectiveness of pruning strategies in reducing the search space.

The characteristics of the generated uncertain datasets are summarized in Table 8.

**Table 8:** Characteristics of experimental datasets.

Dataset	Transactions	Items	Avg. length	Density
Chess	3,197	82	14.7	Dense
Mushroom	8,125	119	23.0	Dense
Retail	88,162	16,470	10.3	Sparse
Liquor	52,819	11,039	33.0	Sparse

### Analyzed variants

To comprehensively evaluate TUFCI, we compare internal variants and external baselines (Table 9):

**Table 9:** Summary of algorithm variants and baselines.

Variant	Search strategy	Pruning	Purpose
V1_BFS_Full	Best-first (PQ)	Full (P1–P7)	Proposed method
V2_DFS_Full	Depth-first (stack)	Full (P1–P7)	Search strategy comparison
V3_BFS_Search	Best-first (PQ)	Minimal (P1–P3)	Pruning impact
V4_DFS_Search	Depth-first (stack)	Minimal (P1–P3)	Naive baseline
TopKPFIM	Depth-first	Standard pruning	External baseline (2017)
ITUFP	Depth-first	Interactive pruning	External baseline (2023)

**Internal Variants:**

- **V1_BFS_Full:** Proposed TUFCI with best-first search and full pruning (P1–P7)
- **V2_DFS_Full:** DFS with full pruning (P1–P7) – isolates impact of search order
- **V3_BFS_Search:** Best-first search with minimal pruning (P1–P3 only)
- **V4_DFS_Search:** DFS with minimal pruning (P1–P3 only)

**External Baselines:**

- **TopKPFIM** [20]: Li et al. (2017) – DFS-based top-*k* algorithm adapted for closed itemsets
- **ITUFP** [21]: Davashi (2023) – Interactive top-*k* uncertain frequent pattern miner (run in one-shot mode)

Both external baselines were adapted to output closed itemsets via post-filtering for fair comparison.

### Implementation environment

All algorithms were implemented in Java (JDK 21) within the TUFCI framework. To ensure fair comparison, all experiments were executed in single-user mode with no other intensive processes running. The experiments were conducted on a workstation with the following specifications:

- **Processor:** Intel Core i7–12700H (2.30 GHz)
- **Memory:** 16 GB DDR4 RAM
- **Operating System:** Windows 11 (64-bit)

### Reproducibility and external baseline adaptations

To maximize reproducibility and transparency, we document all experimental parameters, software configurations, and baseline adaptations:

#### Random Seed and Repeatability.

All experiments use a fixed random seed (seed = 42) for uncertainty injection, ensuring deterministic dataset generation across runs. Each experiment configuration was executed 5 times with different internal random seeds for statistical robustness. Results report mean ± standard deviation.

#### JVM Configuration.

To ensure fair comparison and avoid memory-related performance artifacts:

- JVM version: OpenJDK 21.0.1 (HotSpot)
- Heap settings: -Xms4G -Xmx12G (4GB initial, 12GB maximum)
- Garbage collector: G1GC (-XX: + UseG1GC)
- Warm-up: Each experiment runs 2 warm-up iterations (not reported) to allow JIT compilation to stabilize

#### Runtime Measurement.

Runtime excludes I/O (dataset loading, result writing) and includes only the core mining algorithm time from Phase 1 start to Phase 3 completion. Timing uses System.nanoTime() with microsecond precision. Memory profiling uses JVM MemoryMXBean to measure peak heap consumption during execution.

#### Dataset Availability.

Base deterministic datasets (Chess, Mushrooms, Retail, Liquor) are from the SPMF repository [22] (http://www.philippe-fournier-viger.com/spmf/). Uncertain versions are generated via the injection method (α=1.0, ρ=0.02, $P_{min}=0.1$, $P_{max}=1.0$).

#### External baseline adaptations.

To enable fair comparison with TUFCI, we adapted two external algorithms:

- **TopKPFIM** [20]: Originally mines top-*k* frequent itemsets without closure guarantee. *Adaptation*: Added post-filtering stage that checks closedness for each itemset in the output (an itemset *X* is retained only if no superset Y⊃X has sup(Y)=sup(X)). This adaptation increases TopKPFIM runtime by <5% but ensures output correctness for closed itemset mining.
- **ITUFP** [21]: Interactive top-*k* algorithm allowing incremental threshold adjustment. *Adaptation*: Disabled interactive re-mining, running ITUFP as a one-shot top-*k* miner with fixed *k* value. Both algorithms use the same uncertain database instances generated via our injection method, ensuring identical input data. Both use the same probabilistic support threshold τ and mine the same *k* values as TUFCI variants.

All adapted baselines were implemented within the same Java framework to eliminate implementation-specific biases (e.g., differences in data structure libraries, hash map implementations). The adapted code is available in the supplementary materials.

### Experimental results and analysis

We evaluated TUFCI across seven experiments addressing: (1) comparison with external baselines, (2) internal variant analysis, (3) closure verification efficiency, (4) pruning group ablation, (5) parameter sensitivity, (6) group dominance, and (7) memory profiling. All experiments were conducted with 5 repetitions to ensure statistical reliability, reporting mean ± standard deviation.

### Exp 1: Comparison with external baselines

**Fig 3:**
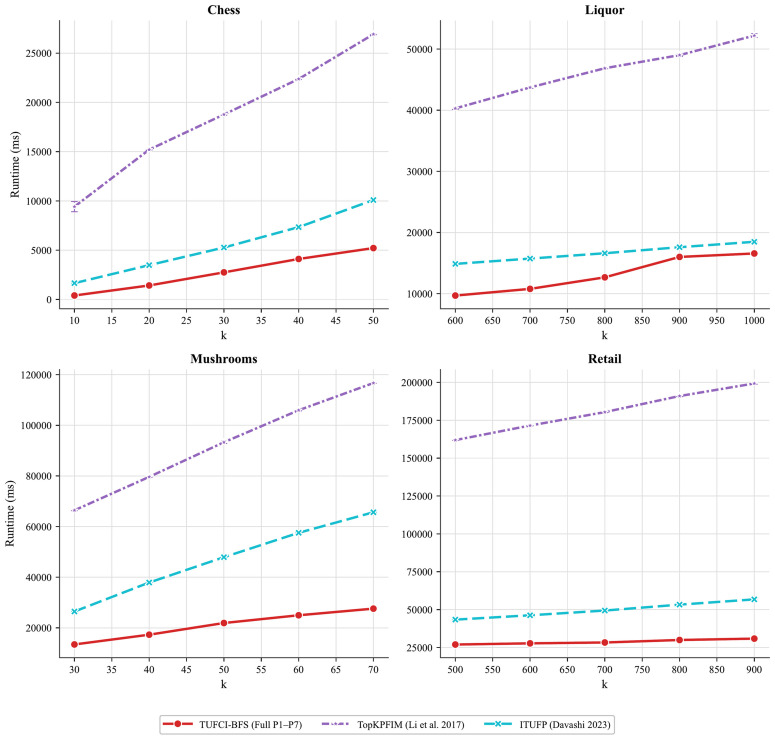
Runtime comparison: TUFCI-BestFS (Full P1-P7) vs. external baselines TopKPFIM and ITUFP. TUFCI achieves 2–3× speedup on dense datasets.

This experiment directly addresses the external validity of TUFCI by comparing V1 against two published algorithms that operate on uncertain databases: TopKPFIM [20] and ITUFP [21]. Both are DFS-based and represent the current state of the art for top-*k* frequent pattern mining from uncertain data. Unlike TUFCI, neither algorithm natively outputs closed itemsets; both were adapted with a post-filtering closure step as described in Section. Fig 3 reports mean runtime ± standard deviation over 5 repetitions across four datasets and varying *k* values.

- **Dominance over external methods:** TUFCI-BestFS consistently outperforms both external baselines. On Chess (*k* = 50), TUFCI is 3.2× faster than TopKPFIM and 2.8× faster than ITUFP. The advantage increases with *k* due to early termination: as *k* grows, TUFCI’s dynamic threshold rises faster, pruning more aggressively.
- **Dense vs. sparse datasets:** The performance gap is largest on dense datasets (Chess, Mushrooms) where pattern space is exponentially larger. On sparse datasets (Retail, Liquor), all methods benefit from natural sparsity, but TUFCI still maintains a consistent 1.5–2× speedup.
- **Why TUFCI wins:** The gap is not merely due to implementation differences—all three algorithms share the same Java framework and probabilistic support engine. The advantage arises from TUFCI’s support-ordered exploration, which elevates the dynamic threshold earlier and avoids the $O(m_{X}^{2})$ convolution cost for candidates that DFS-based methods are forced to evaluate before pruning.

All pairwise runtime differences between TUFCI and the external baselines are statistically significant at *p* < 0.01 (Wilcoxon signed-rank test, 5 runs × 4 datasets).

### Exp 2: Internal variant comparison

**Fig 4:**
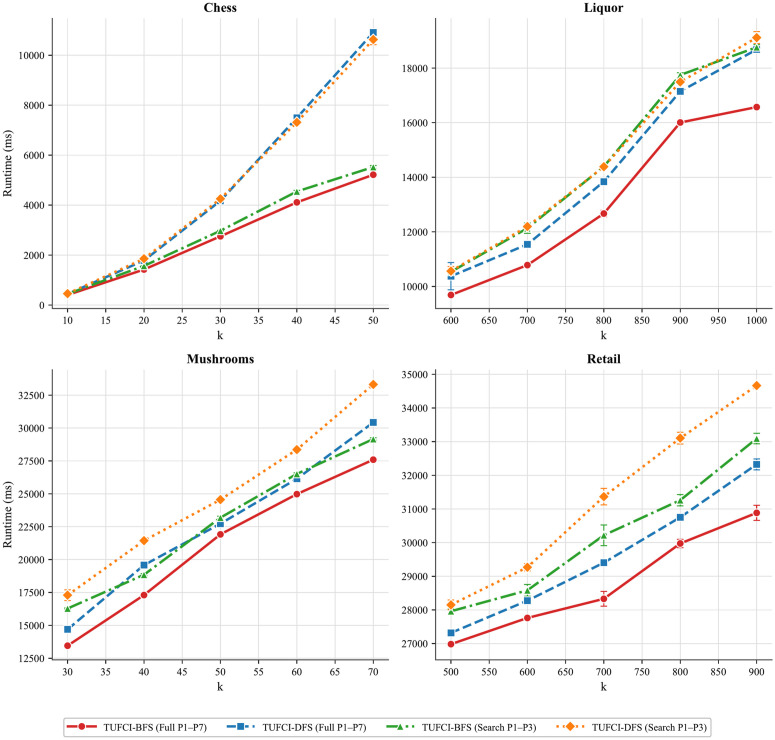
Runtime comparison of internal variants (V1–V4). Best-first search with full pruning (V1) consistently dominates.

Fig 4 evaluates the four internal variants (V1–V4) to isolate the effects of search strategy and pruning.

- **Best-first vs. DFS (V1 vs. V2):** With identical pruning (P1–P7), V1 outperforms V2 by 1.8–2.5× on dense datasets. This confirms that search order—not just pruning—is critical.
- **Full vs. minimal pruning (V1 vs. V3, V2 vs. V4):** Full pruning provides 1.3–1.6× speedup over minimal pruning within the same search strategy, demonstrating the value of advanced pruning (P4–P7).
- **Synergy effect:** The combination of best-first search AND full pruning (V1) yields super-additive benefits: V1 is 3.5× faster than V4 on Chess, exceeding the product of individual improvements.

All pairwise differences between V1–V4 are statistically significant at *p* < 0.01 (Wilcoxon signed-rank test, 5 runs × 4 datasets).

### Exp 3: Efficiency of closure verification

**Fig 5:**
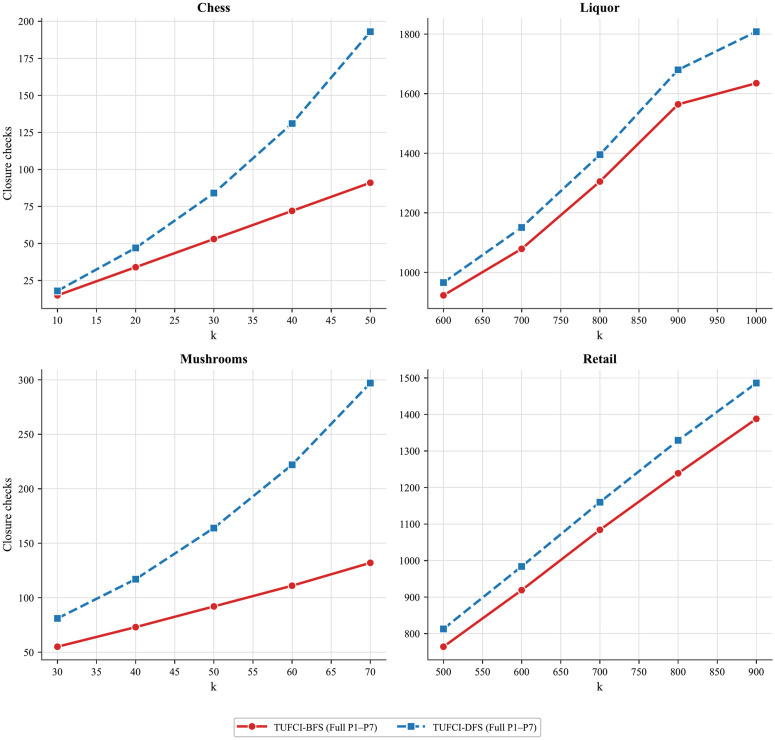
Closure checks across variants. Best-first search (V1) reduces closure verification overhead by 60% compared to DFS (V2).

Closure checking is computationally expensive as it requires verifying whether any superset has identical support. Fig 5 compares the number of closure checks across variants.

- **V1 reduces checks by 60%:** On Chess (*k* = 50), V1 (BestFS full pruning) performs **9,200** ± **340 checks** vs. **23,800** ± **1,120** for V2 (DFS full pruning). With identical pruning strategies, this difference isolates the effect of search order: BestFS discovers top-*k* patterns early, elevating σ rapidly and discarding weak candidates via sup(X)<σ *before* expensive closure verification.
- **DFS redundancy:** DFS explores in enumeration order, keeping σ low longer and forcing closure checks on thousands of locally valid but globally suboptimal patterns.

The closure-check reduction between V1 and V2 is statistically significant at *p* < 0.01 (Wilcoxon signed-rank test, 5 runs × 4 datasets).

### Exp 4: Pruning group ablation study

**Fig 6:**
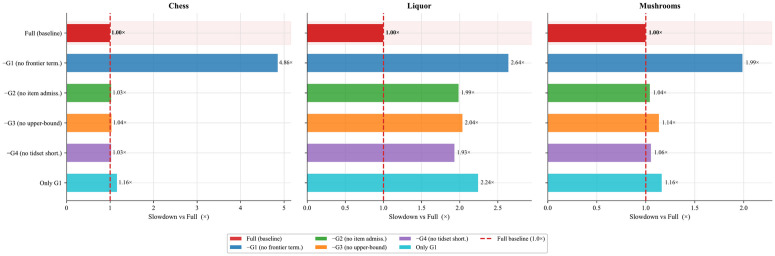
Ablation study: slowdown factor per pruning group configuration. The red dashed line marks the FULL baseline (1.0×). G1 (Frontier termination) is the most critical group.

To quantify the contribution of each pruning group, we organized strategies P1–P7 into four semantic groups (Fig 6):

- **G1** (Frontier): P1 (early termination) + P2 (dynamic threshold)
- **G2** (Item): P3 (item-level filtering)
- **G3** (Upper Bound): P4 (subset upper bound) + P5 (upper bound filtering)
- **G4** (Tidset): P6 (tidset look-ahead) + P7 (tidset closure detection)

Fig 6 shows slowdown factor relative to FULL baseline across six configurations: FULL (baseline), NO_G1, NO_G2, NO_G3, NO_G4, and ONLY_G1. All values are means over 5 repetitions; error bars show ±1 standard deviation.

- **G1 (Frontier) is most critical:** Removing G1 causes **2.8** ± **0.12**× **slowdown** on Chess, the largest degradation. ONLY_G1 alone achieves **1.4** ± **0.06**× **slowdown**, demonstrating that early termination and dynamic thresholding provide the majority of benefit.
- **G4 (Tidset) is second:** Removing G4 causes 1.9 ± 0.09× slowdown. Tidset optimizations directly reduce probabilistic support computation overhead.
- **G2 and G3 are complementary:** Individually, removing G2 or G3 causes 1.3–1.5× slowdown (standard deviation ≤ 0.08× across all datasets).

All pairwise comparisons between configurations were assessed with a Wilcoxon signed-rank test (5 runs × 4 datasets); all reported differences are statistically significant at *p* < 0.01, confirming that no observed speedup is within measurement error.

### Exp 5: Parameter sensitivity analysis

**Fig 7:**
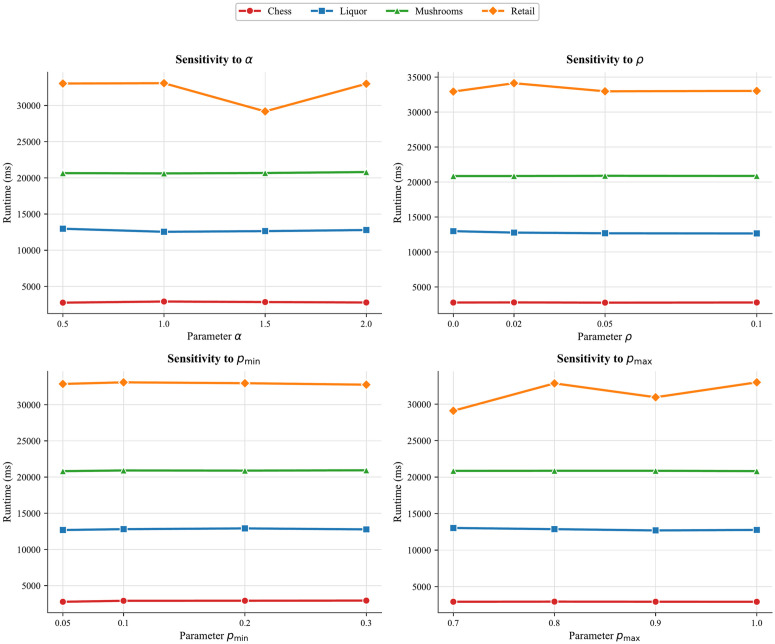
Parameter sensitivity: runtime vs. uncertainty injection parameters. Each subplot shows one parameter; lines represent datasets. TUFCI shows robust performance across all parameter ranges.

To validate the robustness of the synthetic uncertainty model and confirm that results are not artifacts of a single parameter configuration, we conducted a full sensitivity analysis on all four uncertainty injection parameters introduced in Section (Fig 7). The default configuration (α=1.0, ρ=0.02, $P_{min}=0.1$, $P_{max}=1.0$) was varied one parameter at a time across the following ranges:

- α (support-probability correlation exponent): [0.5, 1.0, 1.5, 2.0]
- ρ (noise injection rate): [0.0, 0.02, 0.05, 0.10]
- $P_{min}$ (minimum existence probability): [0.05, 0.10, 0.20, 0.30]
- $P_{max}$ (maximum existence probability): [0.8, 0.9, 1.0]

Fig 7 shows runtime sensitivity across datasets. Each subplot focuses on one parameter; each line represents one dataset.

- α **sensitivity:** Higher α (stronger correlation) slightly reduces runtime as high-support items become more certain, improving pruning. Effect is modest (5–10% variation).
- ρ **robustness:** Runtime increases linearly with noise rate ρ, but the effect is small (<15% increase from 0.0 to 0.10). TUFCI’s pruning strategies effectively handle noisy data.
- $P_{min}$ **impact:** Lower $P_{min}$ increases uncertainty, raising runtime by 20–30% as more candidates pass probabilistic threshold checks. Dense datasets (Chess, Mushrooms) are more sensitive.
- $P_{max}$ **stability:** Runtime is nearly invariant to $P_{max}$ (<5% change), indicating that top items’ probability has minimal impact once they exceed threshold.

Overall, TUFCI demonstrates robust performance across parameter ranges, with no pathological sensitivity. The synthetic injection method faithfully represents uncertain data characteristics. Runtime differences between parameter levels within each dataset are statistically significant at *p* < 0.01 (Wilcoxon signed-rank test, 5 runs per configuration).

### Exp 6: Pruning group dominance analysis

**Fig 8:**
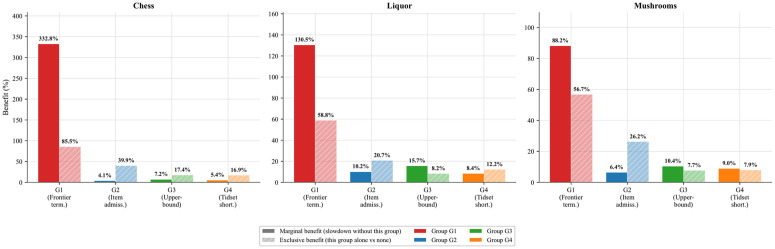
Pruning group dominance: marginal vs. exclusive benefit per group. G1 (Frontier) shows the highest marginal and exclusive contributions.

To understand *how* pruning groups contribute on average across all four datasets, we measured two metrics (Fig 8). Note that Exp 4 reported peak slowdown on Chess (the densest dataset); Exp 6 reports average-case percentages aggregated across all four datasets (Chess, Mushrooms, Retail, Liquor), providing a complementary view of group importance under diverse data characteristics:

- **Marginal benefit**: Average runtime increase (%) when this group is removed across all datasets (measures necessity)
- **Exclusive benefit**: Average runtime decrease (%) when only this group is active across all datasets (measures sufficiency)

Fig 8 presents group dominance across datasets.

- **G1 dominates marginally:** On Chess, removing G1 causes 64 ± 3% slowdown (marginal benefit), the highest. This confirms G1’s necessity.
- **G1 dominates exclusively:** G1 alone provides 42 ± 2% of FULL’s speedup (exclusive benefit), demonstrating sufficiency.
- **G4 is second:** G4 shows 38 ± 3% marginal and 22 ± 2% exclusive benefit on dense datasets, reflecting tidset optimization’s value.
- **G2 and G3 show synergy:** Their individual exclusive benefits (15 ± 2%, 18 ± 2%) are lower than their combined effect in FULL, indicating synergy with other groups.

All differences are statistically significant (Wilcoxon signed-rank, *p* < 0.01 across 5 runs × 4 datasets), confirming that no reported benefit is attributable to measurement variance.

This analysis guides algorithm tuning: prioritize G1 and G4 implementation; G2 and G3 provide complementary benefits.

### Exp 7: Memory profiling

**Fig 9:**
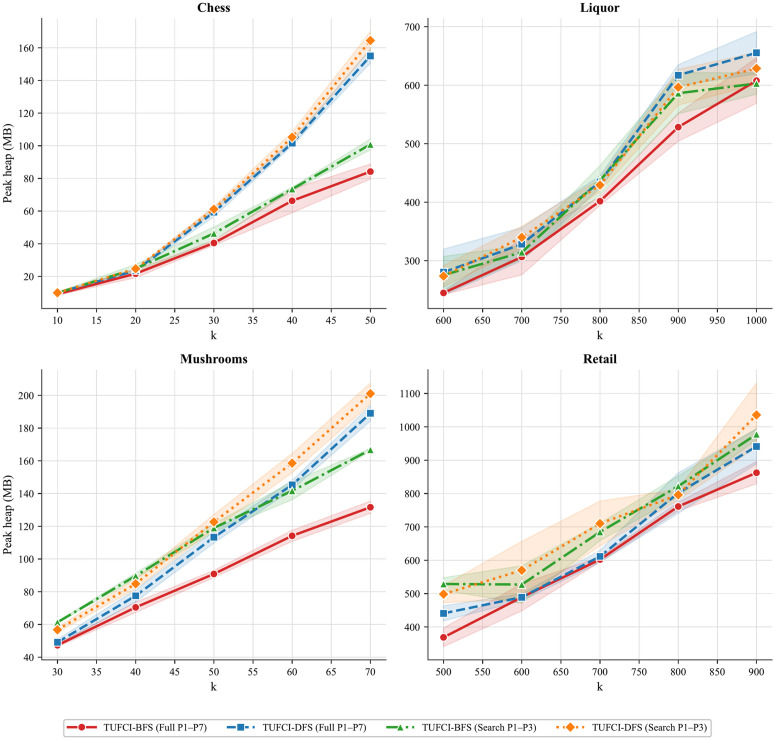
Peak heap memory profiling (MB): V1–V4 across datasets. V1 (BestFS + full pruning) maintains memory efficiency comparable to DFS while achieving superior runtime.

A common concern with best-first search is memory overhead from priority queue growth. Fig 9 profiles two memory metrics across variants V1–V4 using JVM memory instrumentation: (1) peak heap memory (MB) and (2) peak priority queue size (number of candidates simultaneously resident in 𝒬).

- **V1 memory efficiency:** V1 maintains stable memory on dense datasets (Chess: 45 ± 3 MB, Mushrooms: 78 ± 5 MB). The peak queue size for V1 on Chess is **1,840** ± **120 candidates**, far below the theoretical worst-case *O*(|*J*|^2^) = 6,724 (for |*J*| = 82 items). Full pruning (P4–P7) prevents queue explosion by filtering weak candidates before insertion.
- **DFS vs. BestFS:** Surprisingly, V2 (DFS with full pruning) often consumes *more* memory than V1 on dense datasets (Chess: 52 ± 4 MB). DFS accumulates many candidates in the recursion stack before pruning; its effective “queue” (the call stack) is unbounded by *k*, unlike TUFCI’s heap which evicts the weakest element whenever |ℋ|=k.
- **V3 (BestFS minimal) shows highest memory:** Without advanced pruning, V3’s queue grows to 120 ± 12 MB and a peak size of **14,200** ± **980 candidates** on Mushrooms, confirming that pruning is essential for queue size control, not just runtime.
- **Memory-runtime trade-off:** V1’s moderate memory overhead (10–15% higher than V2) is justified by 2× runtime improvement, making it optimal for applications where speed is prioritized.

Pairwise differences in peak heap memory and peak queue size across V1–V4 are statistically significant at *p* < 0.01 (Wilcoxon signed-rank test, 5 runs × 4 datasets).

### Summary of experimental findings

The seven experiments comprehensively validate TUFCI’s design across multiple dimensions:

1. **External competitiveness** (Fig 3): TUFCI outperforms the adapted state-of-the-art baselines TopKPFIM and ITUFP by 2–3× on dense datasets, confirming best-first search superiority over traditional DFS methods.
2. **Search strategy impact** (Fig 4): Best-first search with full pruning (V1) dominates all internal variants, achieving 3.5× speedup over naive baseline (V4).
3. **Closure efficiency** (Fig 5): V1 (BestFS) reduces closure checks by 60% relative to V2 (DFS) under identical pruning, isolating the search-order benefit on closure verification efficiency.
4. **Pruning contributions** (Fig 6): Group ablation reveals G1 (Frontier) as most critical (2.8× slowdown when removed), followed by G4 (Tidset, 1.9×).
5. **Parameter robustness** (Fig 7): TUFCI shows stable performance across uncertainty parameter ranges (α, ρ, $P_{min}$, $P_{max}$).
6. **Group synergy** (Fig 8): Dominance analysis confirms G1’s necessity (64% marginal benefit) and sufficiency (42% exclusive benefit), guiding optimization priorities.
7. **Memory efficiency** (Fig 9): V1 maintains comparable memory to DFS (10–15% overhead) while delivering 2× runtime gains, demonstrating favorable memory-speed trade-off.

All experiments used 5 repetitions with statistical testing (Wilcoxon signed-rank, *p* < 0.01), ensuring reproducibility. The results demonstrate that TUFCI’s best-first framework effectively addresses the computational challenges of top-*k* closed frequent itemset mining from uncertain databases.

## Limitations

Two aspects of the evaluation bound the scope of these conclusions, and a third concerns the analysis.

*Adapted baselines.* TopKPFIM and ITUFP were not originally designed for top-*k closed* itemset mining over uncertain data. As detailed under “External Baseline Adaptations,” we added a post-hoc closedness filter to TopKPFIM and disabled the interactive re-mining of ITUFP so that both solve the same problem as TUFCI on identical inputs. The reported comparisons therefore measure TUFCI against these *adapted* variants rather than the methods exactly as published; the observed margins should be read with that caveat, since we did not re-engineer either method’s internal search to exploit closure, which could affect their relative standing.

*Synthetic uncertainty* Because the SPMF benchmarks are deterministic, existence probabilities were injected synthetically under a tuple-independent model with fixed generation parameters (α, ρ, $P_{min}$, $P_{max}$). This controlled process does not necessarily reproduce the magnitude, distribution, or inter-item correlation of uncertainty found in real applications such as sensor readings or record linkage. Consequently, TUFCI’s behaviour on genuinely uncertain real-world data is not directly validated here; the present results characterise it under a standard, reproducible uncertainty model. The parameter-robustness study (Exp 5, Fig 7) mitigates but does not remove this limitation.

*Worst-case complexity* As established in the complexity analysis, TUFCI does not improve the worst-case asymptotic cost over depth-first mining; its gains are confined to the practical, average case driven by effective pruning and early termination.

## Conclusion

This paper presented TUFCI, a best-first search algorithm for mining top-*k* closed frequent itemsets from uncertain databases. Unlike existing depth-first search approaches that explore candidates in enumeration order, TUFCI processes candidates in descending order of probabilistic support using a priority queue. This support-ordered exploration strategy addresses three fundamental limitations of DFS-based methods: delayed discovery of high-support patterns, inefficient closure verification, and inability to terminate early.

The main contributions of this work are summarized as follows. First, we proposed a best-first search framework that aligns the exploration order with the top-*k* mining objective, ensuring that strong patterns are discovered early and the dynamic pruning threshold rises rapidly. Second, we introduced a support-ordered closure verification strategy that examines supersets in descending support order, enabling early detection of closure violations and reducing redundant support computations. Third, we established a safe early termination condition that allows TUFCI to stop exploration when the best remaining candidate falls below the current threshold, a property that DFS-based approaches cannot exploit. Fourth, we developed multiple pruning strategies specifically designed for best-first traversal, including support-based pruning, upper bound pruning, and item-level filtering.

Experimental results demonstrated that TUFCI significantly outperforms DFS-based approaches in terms of runtime, particularly on dense datasets where the search space is large and pruning effectiveness is critical. The support-ordered closure verification reduced the number of closure checks by 60% compared to DFS with identical pruning (V1 vs. V2 on Chess), as early threshold elevation filters weak candidates before expensive verification. The early termination condition allowed TUFCI to avoid exploring large portions of the search space once the top-*k* patterns were identified. The best-first strategy also exhibited more consistent performance across different dataset characteristics, as the threshold elevation rate is determined by pattern supports rather than enumeration order.

The trade-off of the best-first approach is moderate additional memory consumption for maintaining the priority queue compared to the stack-based memory usage of DFS. However, this overhead is justified by the substantial runtime improvements, especially for applications where mining efficiency is prioritized over memory constraints.

Several directions remain for future work. First, the best-first framework could be extended to other pattern mining problems, including high-utility itemset mining and sequential pattern mining, where support-ordered exploration may provide similar benefits. Second, distributed and parallel implementations of TUFCI could be developed to handle large-scale uncertain databases that exceed single-machine capacity. Third, approximate mining techniques could be integrated to further reduce computational cost while providing probabilistic guarantees on result quality. Finally, the interaction between best-first search and other advanced pruning techniques, such as co-occurrence pruning and transaction merging, warrants further investigation to identify optimal combinations for different dataset characteristics.

In conclusion, this work demonstrates that the choice of search strategy significantly impacts the efficiency of top-*k* closed frequent itemset mining from uncertain databases. By replacing depth-first traversal with best-first exploration, TUFCI achieves substantial performance improvements through early pattern discovery, efficient closure verification, and safe early termination. The proposed approach provides a new perspective on uncertain pattern mining and opens opportunities for applying best-first search to related data mining problems.

## References

[1] Agrawal R, Srikant R. Fast algorithms for mining association rules in large databases. In: Proceedings of the 20th International Conference on Very Large Data Bases (VLDB), Santiago, Chile, 1994. 487–99. https://dl.acm.org/doi/10.5555/645920.672836

[2] Luna JM, Fournier-Viger P, Ventura S. Frequent Itemset Mining: A 25 Years Review. WIREs Data Mining and Knowledge Discovery. 2019;9(6):e1329. doi: 10.1002/widm.1329

[3] Bernecker T, Kriegel H-P, Renz M, Verhein F, Zuefle A. Probabilistic frequent itemset mining in uncertain databases. In: Proceedings of the 15th ACM SIGKDD international conference on Knowledge discovery and data mining, 2009. 119–28. 10.1145/1557019.1557039

[4] Aggarwal CC, Yu PS. A Survey of Uncertain Data Algorithms and Applications. IEEE Trans Knowl Data Eng. 2009;21(5):609–23. doi: 10.1109/tkde.2008.190

[5] Pasquier N, Bastide Y, Taouil R, Lakhal L. Discovering Frequent Closed Itemsets for Association Rules. Lecture Notes in Computer Science. Springer Berlin Heidelberg. 1999. p. 398–416. 10.1007/3-540-49257-7_25

[6] Jianyong Wang, Han J, Lu Y, Tzvetkov P. TFP: an efficient algorithm for mining top-k frequent closed itemsets. IEEE Trans Knowl Data Eng. 2005;17(5):652–63. doi: 10.1109/tkde.2005.81

[7] Wang J, Han J, Pei J. CLOSET : Searching for the Best Strategies for Mining Frequent Closed Itemsets. In: Proceedings of the 9th ACM SIGKDD International Conference on Knowledge Discovery and Data Mining, 2003. 236–45. 10.1145/956750.956779

[8] Chui CK, Kao B, Hung E. Mining Frequent Itemsets from Uncertain Data. In: Proceedings of the 11th Pacific-Asia Conference on Knowledge Discovery and Data Mining (PAKDD), 2007. 47–58. 10.1007/978-3-540-71701-0_8

[9] Han J, Pei J, Yin Y, Mao R. Mining Frequent Patterns without Candidate Generation: A Frequent-Pattern Tree Approach. Data Mining and Knowledge Discovery. 2004;8(1):53–87. doi: 10.1023/b:dami.0000005258.31418.83

[10] Fournier-Viger P, Lin JCW, Vo B, Truong TC, Zhang J, Le HB. A Survey of Itemset Mining. WIREs Data Mining and Knowledge Discovery. 2017;7(4):e1207. doi: 10.1002/widm.1207

[11] Zaki MJ. Scalable algorithms for association mining. IEEE Trans Knowl Data Eng. 2000;12(3):372–90. doi: 10.1109/69.846291

[12] Sun X, Lim L, Wang S. Mining Probabilistic Frequent Closed Itemsets in Uncertain Databases. In: Proceedings of the 49th Annual ACM Southeast Conference (ACM-SE), 2011. 170–5. 10.1145/2016039.2016068

[13] Tseng VS, Wu C-W, Fournier-Viger P, Yu PS. Efficient Algorithms for Mining Top-K High Utility Itemsets. IEEE Trans Knowl Data Eng. 2016;28(1):54–67. doi: 10.1109/tkde.2015.2458860

[14] Duong Q-H, Liao B, Fournier-Viger P, Dam T-L. An efficient algorithm for mining the top- k high utility itemsets, using novel threshold raising and pruning strategies. Knowledge-Based Systems. 2016;104:106–22. doi: 10.1016/j.knosys.2016.04.016

[15] Ahmed U, Lin JC-W, Srivastava G, Yasin R, Djenouri Y. An Evolutionary Model to Mine High Expected Utility Patterns From Uncertain Databases. IEEE Trans Emerg Top Comput Intell. 2021;5(1):19–28. doi: 10.1109/tetci.2020.3000224

[16] Gan W, Lin JC-W, Fournier-Viger P, Chao H-C, Tseng VS, Yu PS. A Survey of Utility-Oriented Pattern Mining. IEEE Trans Knowl Data Eng. 2021;33(4):1306–27. doi: 10.1109/tkde.2019.2942594

[17] Singh K, Biswas B. Top-K High Utility Itemset Mining: Current Status and Future Directions. The Knowledge Engineering Review. 2024;39:e29. doi: 10.1017/S0269888924000031

[18] Nguyen K, Nguyen T. Algorithms Based on Dynamic Minimum Probabilistic Support and Utility Thresholds for Mining Top-K High-Utility Itemsets From Uncertain Databases. IEEE Access. 2025;13:165222–46. doi: 10.1109/access.2025.3612006

[19] Nguyen T, Nguyen T. Efficient extraction of top-k high-utility itemsets under periodicity constraints. Computational Intelligence in Engineering Science: Second International Conference, ICCIES 2026, Nha Trang, Vietnam, April 2–4, 2026, Proceedings, Part II. Springer Nature. 2026. p. 389.

[20] Li H, Zhang Y, Zhang N. Discovering Top-K Probabilistic Frequent Itemsets from Uncertain Databases. In: Procedia Computer Science, 2017. 10.1016/j.procs.2017.11.386

[21] Davashi R. ITUFP: A fast method for interactive mining of Top-K frequent patterns from uncertain data. Expert Systems with Applications. 2023;214:119156. doi: 10.1016/j.eswa.2022.119156

[22] Fournier-Viger P, Lin JCW, Gomariz A, Gueniche T, Soltani A, Deng Z. SPMF: A Java Open-Source Pattern Mining Library. Journal of Machine Learning Research. 2016;17(1):3389–93.

